# BMP restricts stemness of intestinal Lgr5^+^ stem cells by directly suppressing their signature genes

**DOI:** 10.1038/ncomms13824

**Published:** 2017-01-06

**Authors:** Zhen Qi, Yehua Li, Bing Zhao, Chi Xu, Yuan Liu, Haonan Li, Bingjie Zhang, Xinquan Wang, Xiao Yang, Wei Xie, Baojie Li, Jing-Dong Jackie Han, Ye-Guang Chen

**Affiliations:** 1 The State Key Laboratory of Membrane Biology, Tsinghua-Peking Center for Life Sciences, School of Life Sciences, Tsinghua University, Beijing 100084, China; 2 Chinese Academy of Sciences Key Laboratory of Computational Biology, Chinese Academy of Sciences-Max Planck Partner Institute for Computational Biology, Shanghai Institutes for Biological Sciences, Chinese Academy of Sciences, Shanghai 200031, China; 3 Graduate University of Chinese Academy of Sciences, Beijing 100049, China; 4 Ministry of Education Key Laboratory of Protein Science, Center for Structural Biology, School of Life Sciences, Tsinghua University, Beijing 100084, China; 5 State Key Laboratory of Proteomics, Genetic Laboratory of Development and Diseases, Institute of Biotechnology, Beijing 100071, China; 6 Bio-X Institutes, Key Laboratory for the Genetics of Developmental and Neuropsychiatric Disorders, Ministry of Education, Shanghai Jiao Tong University, Shanghai 200240, China

## Abstract

The intestinal epithelium possesses a remarkable self-renewal ability, which is mediated by actively proliferating Lgr5^+^ stem cells. Bone morphogenetic protein (BMP) signalling represents one major counterforce that limits the hyperproliferation of intestinal epithelium, but the exact mechanism remains elusive. Here we demonstrate that epithelial BMP signalling plays an indispensable role in restricting Lgr5^+^ stem cell expansion to maintain intestinal homeostasis and prevent premalignant hyperproliferation on damage. Mechanistically, BMP inhibits stemness of Lgr5^+^ stem cells through Smad-mediated transcriptional repression of a large number of stem cell signature genes, including *Lgr5*, and this effect is independent of Wnt/β-catenin signalling. Smad1/Smad4 recruits histone deacetylase HDAC1 to the promoters to repress transcription, and knockout of Smad4 abolishes the negative effects of BMP on stem cells. Our findings therefore demonstrate that epithelial BMP constrains the Lgr5^+^ stem cell self-renewal via Smad-mediated repression of stem cell signature genes to ensure proper homeostatic renewal of intestinal epithelium.

## Abstract (web summary)

Bone morphogenetic protein (BMP) maintains intestinal homeostasis by restricting its hyperproliferation but whether it directly regulates the stem cells is unknown. Here the authors show that BMP constrains the Lgr5^+^ stem cell expansion under both homeostatic and injury conditions through Smad-mediated repression of stem cell signature genes.

The intestinal epithelium is characterized by its remarkable self-renewal ability, with a turnover time of 4–5 days, and the crypt base columnar cells marked by Lgr5 represent the actively proliferating stem cells that mediate the daily renewal of intestinal epithelium^1^,^2^,^3^. This fast renewal kinetics of intestinal epithelium puts it at a high risk of hyperproliferation and thus tumorigenesis, therefore its homeostasis is tightly controlled by both positive and negative signals^4^. Among these signals, bone morphogenetic protein (BMP) plays a pivotal role in balancing the Wnt-driven homeostatic self-renewal and proliferation of intestinal epithelium^5^,^6^,^7^. BMPs belong to the transforming growth factor-β (TGF-β) superfamily of ligands, and like other members of this superfamily, binding of BMPs to their type II receptors leads to phosphorylation and thus activation of the type I receptors (BMPRI). BMPRI then phosphorylates and activates R-Smads (Smad1, 5 and 8), allowing them to form complexes with Co-Smad (Smad4) and translocate into the nucleus to regulate gene expression^8^,^9^,^10^,^11^. BMP receptors can also signal through non-Smad pathways such as p38, JNK and PI3K to regulate multiple cellular functions^12^,^13^. In intestine, BMP2 and BMP4 ligands are secreted by intravillus and intercrypt mesenchymal cells^14^,^15^,^16^, while the BMP antagonists Noggin and Gremlin 1 are mainly expressed in mesenchymal cells beneath the crypt, resulting in the formation of a gradient of BMP activity along the crypt–villus axis^17^,^18^. It has long been recognized that BMP signalling restricts intestinal epithelium hyperproliferation. Frequent germline mutations in the genes encoding the type I BMP receptor *BMPRIA* or the downstream signal mediator *SMAD4* have been found in patients with juvenile polyposis syndrome, an inherited hamartomatous polyposis disorder with an increased risk of colorectal cancer initiation^19^,^20^,^21^. Transgenic overexpression of the BMP antagonist Noggin in intestine or induced whole-body deletion of *Bmpr1a* can drive hyperproliferative intestinal polyposis, resembling the phenotypes of juvenile polyposis syndrome^14^,^17^, and transgenic expression of Gremlin 1 also initiates intestinal tumorigenesis^22^. However, the mechanism underlying the negative regulation of BMP on intestinal self-renewal remains unclear, limiting the therapeutic potentials in targeting this signaling to treat colorectal cancer.

Wnt signalling is essential for the homeostatic self-renewal and proliferation of intestinal stem cell compartment^23^. It was previously reported that BMP negatively regulates intestinal homeostasis by blocking the nuclear accumulation of β-catenin via PTEN-dependent suppression of Akt activity in BrdU-retaining +4 quiescent stem cells^17^. However, this model needs further validation as high Wnt/β-catenin transcriptional activity is mainly present in Lgr5^+^ intestinal stem cells (ISCs) and transient amplifying cells, while +4 quiescent stem cells are insensitive to Wnt signalling^5^,^24^,^25^,^26^,^27^,^28^. It is proposed that the +4 quiescent stem cells are involved in injury repair on irradiation but do not mediate the daily intestinal renewal^27^,^29^. In contrast, actively cycling Lgr5^+^ ISCs are responsible for this daily renewal, but little is known about whether BMP regulates the intestinal renewal during normal homeostasis through influencing the Lgr5^+^ ISCs. Although several studies have suggested that BMP signalling might inhibit Lgr5^+^ stem cell activity in *in vitro*-cultured organoids and intestinal cancer models^30^,^31^,^32^, it is unclear whether BMP regulates Lgr5^+^ stem cells to maintain intestine epithelium homeostasis *in vivo*. Further, the underlying mechanism has not been explored. In this study, using knockout (KO) mouse models, we demonstrate that BMP restricts Lgr5^+^ stem cell expansion by limiting its self-renewal, and thus prevents epithelial hyperproliferation. This effect is mediated by Smad-dependent transcriptional regulation of a stem cell signature gene programme, but not inhibition of the nuclear translocation of β-catenin in Lgr5^+^ stem cells. Our data unravel a novel mechanism underlying the negative regulation of BMP on ISC expansion to maintain proper intestinal homeostasis.

## Results

### BMP restricts Lgr5^+^ stem cell expansion during homeostasis

**Figure 1:**
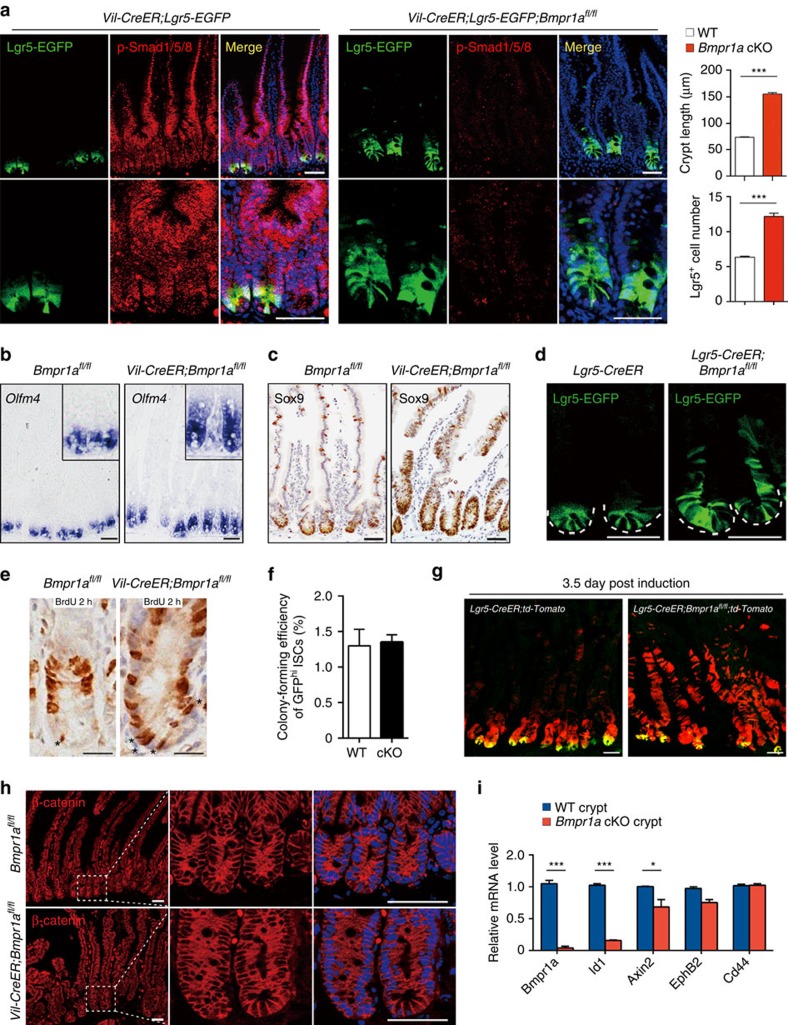
BMP restricts Lgr5^+^ stem cell expansion independently of Wnt/β-catenin during intestinal homeostasis. (**a**) *Vil-CreER;Lgr5-EGFP* and *Vil-CreER*;*Lgr5-EGFP;Bmpr1a*^*fl/fl*^ mice were analysed 1 week after 5-day tamoxifen administration. Proximal jejunum sections were stained for p-Smad1/5/8 (for BMP signalling activity) and EGFP (for stem cells). Nuclei were counter-stained with 4′,6-diamidino-2-phenylindole (DAPI). The lower panels show enlargements of the upper panels. Images are representative of *n*=6 mice per genotype. Quantifications of crypt length and Lgr5^+^ cell number is shown aside, and the data represent mean±s.e.m. of *n*=6 mice per genotype. ***P<0.001 by Student's *t*-tests. (**b**,**c**) *In situ* hybridization of *Olfm4* and immunohistochemical staining of Sox9 in *Bmpr1a*^*fl/fl*^ and *Vil-CreER;Bmpr1a*^*fl/fl*^ mice at day 12. Images are representative of *n*=6 mice per genotype. (**d**) Immunofluorescence images showing Lgr5^+^ stem cells 12 days after the first injection in *Lgr5-CreERT2;Bmpr1a*^*fl/fl*^ mice (*n*=5 mice per genotype). Dotted lines delineate the normal Lgr5^+^ stem cell pool. Images are representative of three independent experiments. (**e**) BrdU incorporation in ISCs and transient amplifying cells after a 2 h pulse in *Vil-CreER;Bmpr1a*^*fl/fl*^ and *Bmpr1a*^*fl/fl*^ mice at day 12. Images are representative of *n*=6 mice per genotype. The asterisks indicate the replicating CBC cells at the base of crypt. (**f**) *In vitro* colony formation assay of GFP^high^ cells sorted from *Vil-CreER;Bmpr1a*^*fl/fl*^ and *Bmpr1a*^*fl/fl*^ mice at day 12. Data represent mean±s.e.m. of *n*=3 experiments. (**g**) Lineage tracing in the jejunum of *Lgr5-CreERT2;td-Tomato* mice and *Lgr5-CreERT2;td-Tomato;Bmpr1a*^*fl/fl*^ mice. Representative images of td-Tomato immunofluorescence at day 3.5 after induction were shown (*n*=4 mice per genotype). (**h**) β-catenin immunofluorescence staining of proximal jejunum sections from *Bmpr1a*^*fl/fl*^ and *Vil-CreER;Bmpr1a*^*fl/fl*^ mice at day 12. The right panels show enlargements of boxed areas. Nuclei were counter-stained with DAPI. Images are representative of *n*=6 mice per genotype. (**i**) Quantitative RT–PCR analysis of *Bmpr1a*, *Id1*, *Axin2*, *EphB2* and *Cd44* expression in intestinal crypts from *Vil-CreER;Bmpr1a*^*fl/fl*^ and control mice at day 12 after induction. Data represent mean±s.e.m. of *n*=5 mice per genotype. *P<0.05, ***P<0.001 by Student's *t*-tests. Scale bars, 50 μm. CBC, crypt columnar cell; EGFP, enhanced green fluorescent protein.

It is unclear whether BMP limits the over-expansion of intestinal epithelium through regulating the self-renewal of Lgr5^+^ ISCs or just inhibiting the proliferation of epithelial cells. To address this issue, we assessed the function of the BMP type I receptor Bmpr1a in intestinal epithelium by crossing *Bmpr1a*^*fl/fl*^ mice with the inducible *Villin-CreERT2* (*Vil-CreER*) mice. Five-day tamoxifen administration led to nearly complete ablation of Bmpr1a in whole intestinal epithelium within 12 days after the first injection (Supplementary Fig. 1a–c). The mutant mice exhibited rapid weight loss and intestinal distention, especially in duodenum and jejunum (Supplementary Fig. 1d,e), suggesting defective intestine hyperplasia due to hyperactive stem cells in the mutant mice. Indeed, loss of BMP activity at day 12 after tamoxifen administration in *Vil-CreER;Lgr5-EGFP;Bmpr1a*^*fl/fl*^ mice was accompanied by a strong expansion of Lgr5^+^ ISCs towards the upper part of the crypts (Fig. 1a). The expansion of the stem cells was supported by the profound increase in the number of Olfm4-positive and Sox9-positive cells (Fig. 1b,c). We observed that relatively low but clear BMP signaling activity in Lgr5^+^ ISCs at the bottom of crypts, as shown by phospho-Smad1/5/8 staining before and after Bmpr1a inactivation (Fig. 1a). As loss of BMP signalling resulted in a marked expansion of stem cells, these data suggest that the low level of BMP signalling activity in Lgr5^+^ ISCs is important to restrict their stemness during homeostasis. In addition, the number of Paneth cells was increased, indicating that the stem cell niche was subsequently enlarged (Supplementary Fig. 1f), and the turnover rate of epithelial cells was accelerated significantly after *Bmpr1a* inactivation (Supplementary Fig. 1g). To examine whether loss of BMP response in Lgr5^+^ ISCs is responsible for their expansion, we deleted *Bmpr1a* specifically in these cells using *Lgr5-EGFP-IRES-CreERT2;Bmpr1a*^*fl/fl*^ (*Lgr5-CreER;Bmpr1a*^*fl/fl*^) mice. Indeed, ISC-specific *Bmpr1a* deletion also resulted in the apparent expansion of Lgr5^+^ ISCs (Fig. 1d).

### Functional stem cells are increased without Wnt activation

To investigate whether functional stem cells are increased in *Bmpr1a* cKO mice, we assessed incorporation of 5-bromodeoxyuridine (BrdU) into ISCs (crypt base columnar cells) and transient amplifying cells. After a short-term (2 h) pulse, BrdU incorporation into the crypt base columnar cells was remarkably increased in the *Bmpr1a* cKO mice. This indicates that loss of BMP augmented replicating ISCs number due to an increase in the total number of ISCs (Fig. 1e). Importantly, the *in vitro* colony formation assays demonstrate that all the Lgr5 high cells in the *Bmpr1a* cKO mice possesses equal ability to form organoids compared with the cells from control mice (Fig. 1f). These results suggest that the number of Lgr5 high cells was increased in the *Bmpr1a* cKO mice and all these cells were functional stem cells. It is worth noting that the presence of Noggin in the *in vitro* single-cell colony formation system eliminates the difference in the self-renewal ability between wild-type and Bmpr1a-loss Lgr5-high cells. We further confirmed the functionality of the expanded stem cells in *Bmpr1a* cKO mice by the cell tracing experiment. *Lgr5-CreER;Bmpr1a*^*fl/fl*^ mice were crossed with *ROSA26 td-Tomato* reporter mice, and at day 3.5 following tamoxifen induction, Tomato-positive cells extended markedly towards the villus in *Lgr5-CreER;Bmpr1a*^*fl/fl*^ mice, while most Tomato-positive cells were still localized in the crypts in control mice (Fig. 1g). These results indicate that the stem cell function of Lgr5^+^ cells was boosted on loss of BMP activity during homeostasis.

Interestingly, we never observed nuclear accumulation of β-catenin in the hyperplastic crypts from the mutant mice (Fig. 1h), which is in contrast to the obvious nuclear accumulation of β-catenin in the transformed crypts on *Apc* loss (Supplementary Fig. 1h), suggesting that BMP does not directly inhibit the nuclear translocation of β-catenin. Consistent with this, the expression of Wnt targets, such as *Axin2*, *EphB2* and *Cd44*, was not increased in the mutant crypts while canonical BMP target *Id1* was dramatically decreased (Fig. 1i).

To examine whether the altered Lgr5^+^ ISC expansion has a long-term effect on intestine homeostasis, we examined the intestine with impaired BMP signalling for longer time. In the *Bmpr1a* deletion mice after 1.5 months, the intestine was enlarged in size, with dilated crypts, proliferative villus epithelium invaginations and moderately expanded stromal tissue (Supplementary Fig. 2a–d), resembling the early phenotypes in juvenile polyposis syndrome. Lgr5^+^ ISCs were markedly expanded in the mutant mice, and this enlarged Lgr5^+^ stem cell pool could even promote the formation of microadenoma-like abnormal structure in the crypt compartment, but no stem cell property was detected in villus invaginations (Supplementary Fig. 2e,f), further demonstrating that hyperactive Lgr5^+^ ISCs in crypts are responsible for the intestinal hyperplasia phenotype. Of note, Wnt/β-catenin signalling remained unchanged in the mutant intestine (Supplementary Fig. 2g). After 3 months, we detected polyps in the intestine from *Vil-CreER;Lgr5-CreER;Bmpr1a*^*fl/fl*^ mice (Supplementary Fig. 2h–j). The polyps were similar to previous reported ones in Noggin transgenic mice or *Bmpr1a* KO mice^14^,^17^. Therefore, our data demonstrate that epithelial BMP signalling plays a critical role in preventing the over-expansion of Lgr5^+^ stem cells and subsequent epithelium outgrowth in the physiological condition.

### Loss of BMP response accelerates crypt regeneration

**Figure 2:**
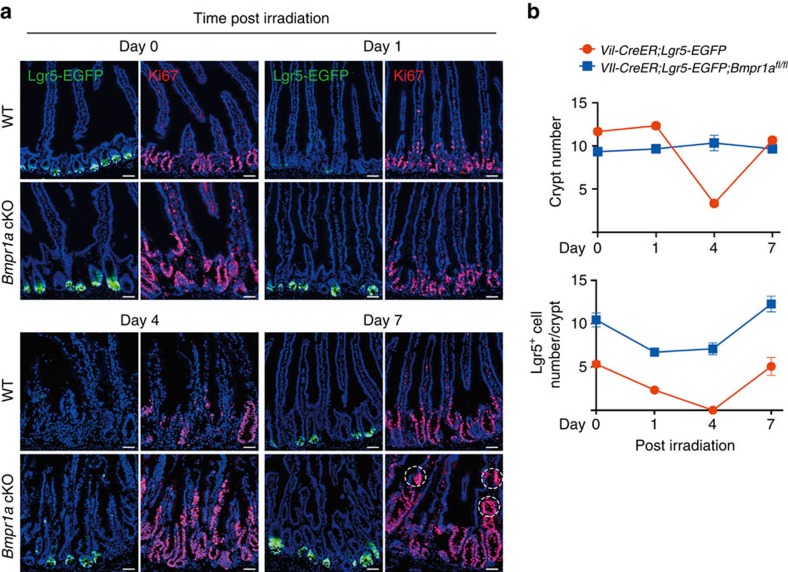
Loss of BMP accelerates crypt regeneration and drives premalignant hyperproliferation after exposure to ionizing radiation. (**a**) Representative images of Lgr5-EGFP immunofluorescence and Ki67 staining in jejunum sections from *Lgr5-EGFP* and *Vil-CreER;Lgr5-EGFP;Bmpr1a*^*fl/fl*^ mice at different time points after 10 Gy abdominal X-ray radiation (*n*=5 mice per genotype). The mice were irradiated 2 weeks following 5-day tamoxifen induction. Dotted lines delineate the proliferating epithelial invaginations on villus. (**b**) Quantification of crypt number and Lgr5^+^ stem cell number in *Lgr5-EGFP* and *Vil-CreER;Lgr5-EGFP;Bmpr1a*^*fl/fl*^ mice at different time points after 10 Gy abdominal X-ray radiation. Data represent mean±s.e.m. of *n*=5 mice per genotype. Scale bars, 50 μm.

Given that Lgr5^+^ ISC numbers increased in *Bmpr1a* cKO mice, we asked whether loss of BMP response accelerates intestinal regeneration after irradiation. The intestinal epithelium is sensitive to DNA damage caused by ionizing radiation, but can efficiently recover from low doses of radiation^33^,^34^. Lgr5^+^ stem cells are hypersensitive to DNA damage caused by irradiation, but they also has a higher capacity for DNA repair compared with other cells in the crypt^35^. Consistent with early findings^35^,^36^,^37^, we observed that irradiation with 10 Gy X-ray caused loss of Lgr5^+^ stem cells at day 1, followed by the massive loss of crypts at day 4 in control mice (Fig. 2a). Afterwards, the remaining Lgr5^+^ stem cells efficiently proliferate to repopulate the crypt, and reached to the normal number when the regeneration was nearly completed at day 7 (Fig. 2a). However, a significant number of Lgr5^+^ stem cells were retained in *Bmpr1a* cKO mice after irradiation, and a remarkably accelerated regeneration response was also observed (Fig. 2a,b). Irradiation-induced apoptosis was not affected by loss of BMP signalling, and a lethal dose of irradiation (14 Gy) could efficiently eliminate the Lgr5^+^ stem cells in *Bmpr1a* cKO mice (Supplementary Fig. 3). Interestingly, abnormal hyperproliferation of crypts and occurrence of proliferating epithelial invaginations on villus were detected in the *Bmpr1a* KO at day 7 (Fig. 2a), which is similar to the typical phenotype observed 1.5 months after *Bmpr1a* KO (Supplementary Fig. 2d,e). This indicates that the tumorigenic ability of Lgr5^+^ stem cells was boosted on irradiation-induced damage in the *Bmpr1a* KO mice. These results highlight the essential roles of BMP signalling in guarding the intestinal homeostasis and regeneration.

### BMP suppresses stem cell signature genes in Lgr5^+^ ISCs

**Figure 3:**
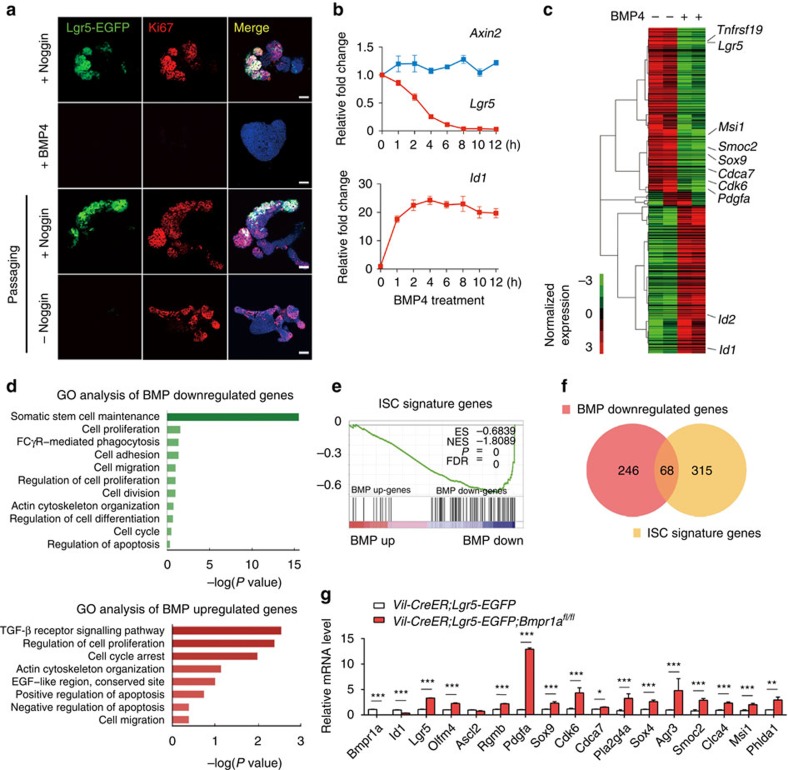
BMP directly suppresses the expression of Lgr5^+^ stem cell signature genes. (**a**) BMP signalling was activated for 36 h in intestinal organoids through direct BMP4 treatment or Noggin withdrawal from the normal culture medium containing EGF, Noggin and R-spondin after cell passaging. Lgr5^+^ stem cells (Lgr5-EGFP) and proliferating cells (Ki67^+^) were examined in the organoids. Nuclei were counter-stained with 4′,6-diamidino-2-phenylindole. Images are representative of three independent experiments. (**b**) Quantitative RT–PCR analysis of *Lgr5*, *Axin2* and *Id1* in intestinal organoids after BMP stimulation for indicated time. (**c**) The organoids derived from *Lgr5-EGFP* mice were treated with 10 ng ml^−1^ BMP4 for 4 h, and then Lgr5^+^ stem cells were isolated by FACS and subjected to RNA-seq. Heatmap of RNA-seq data shows the downregulation of known ISC marker genes by BMP. (**d**) GO analysis of BMP-downregulated genes and BMP-upregulated genes in Lgr5^+^ stem cells, respectively. (**e**) GSEA revealed significant enrichment of ISC signature gene set with BMP-downregulated genes. ES, enrichment score; NES, normalized enrichment score. (**f**) Venn diagram showing the overlap between ISC signature genes with BMP-downregulated genes. (**g**) Quantitative RT–PCR analysis of stem cell signature gene expression in Lgr5^+^ stem cells sorted from mice with indicated genotypes at day 12. Data represent mean±s.e.m of *n*=5 mice. *P<0.05, **P<0.01, ***P<0.001 by Student's *t*-tests. Scale bars, 50 μm.

To understand how BMP restricts the Lgr5^+^ stem cell expansion, we took advantage of the *in vitro* culture system of intestinal organoids or Lgr5^+^ single cells, which needs epidermal growth factor (EGF), R-spondin1 and Noggin^30^. BMP4 led to a rapid loss of Lgr5-GFP cells in the cultured intestinal organoids within 36 h, followed by an obvious growth inhibition (Fig. 3a and Supplementary Fig. 4a). Intriguingly, we observed that low BMP activity resulted from Noggin withdrawal during passaging also caused a rapid loss of Lgr5^+^ ISCs while only having a slight impact on proliferation of transient amplifying cells within 36 h (Fig. 3a and Supplementary Fig. 4b), indicating that Lgr5^+^ ISCs are hypersensitive to BMP. Lineage tracing of recombined Lgr5^+^ stem cells confirmed that the proliferation of Lgr5^+^ stem cells was remarkably decreased on Noggin withdrawal (Supplementary Fig. 4c). Consistently, the expression of stem cell marker genes, including *Lgr5*, *Olfm4* and *Ascl2* was markedly decreased by Noggin withdrawal and BMP addition (Supplementary Fig. 4d–f). Notably, BMP4 induced a decrease of *Lgr5* expression along the time, while having no apparent effect on the expression of *Axin2*, a sensitive Wnt target in the cultured organoids (Fig. 3b). This was contrary to the effect of R-spondin1, whose withdrawal led to a decreased expression of both *Axin2* and *Lgr5* within 4 h (Supplementary Fig. 4g), suggesting that Wnt signalling remained largely normal on BMP activation. As a control, the expression of the BMP target *Id1* was rapidly induced by BMP4 (Fig. 3b).

The above data suggest that BMP restricts the stemness of Lgr5^+^ ISCs possibly through direct regulation of gene expression rather than through inhibition of Wnt/β-catenin signalling. To identify the BMP target genes that mediate the negative effect of BMP on Lgr5^+^ ISCs, the Lgr5-GFP^high^ cells in intestinal organoids were treated with 10 ng ml^−1^ BMP4 for 4 h and then sorted by fluorescence-activated cell sorting (FACS). RNA sequencing (RNA-seq) revealed that BMP4 induced a significant downregulation of many known ISC marker genes in Lgr5^+^ ISCs, including *Tnfrsf19*, *Lgr5*, *Msi1*, *Smoc2*, *Sox9* and *Pdgfa* in 4 h (Fig. 3c and Supplementary Data Set 1). Gene Ontology (GO) analysis further highlighted that BMP downregulated the genes that mediate somatic stem cell maintenance, while upregulating the genes that are involved in cell cycle arrest (Fig. 3d). These observations prompted us to test whether BMP restricts stemness of Lgr5^+^ ISCs through direct transcriptional suppression of a stem cell gene signature programme. We compared our RNA-seq data with a previously identified Lgr5^+^ stem cell signature gene set^38^, and found a significant enrichment of the signature genes in the BMP-downregulated genes (Fig. 3e), and about 18% (68/384) genes displayed a fast reduction of their expression by BMP (Fig. 3f). The transcription factor *Ascl2* is a Wnt target gene and acts as a master regulator of Lgr5^+^ stem cells by directly activating the expression of many stem cell signature genes, including *Lgr5* (refs 26, 39). Notably, our results excluded *Ascl2* as a direct BMP target gene, further supporting the note that BMP directly represses the expression of stem cell signature genes, rather indirectly through controlling some master regulators.

To confirm that BMP-mediated repression of the ISC signature genes *in vivo*, we analysed the gene expression in Lgr5^+^ stem cells from the *Vil-CreER;Lgr5-EGFP;Bmpr1a*^*fl/fl*^ mice. Indeed, 15 out of 25 tested signature genes displayed significant upregulated expression in Lgr5^+^ stem cells of the mutant mice (Fig. 3g). *In situ* hybridization of the signature gene *Pdgfa* also confirmed the result (Supplementary Fig. 4h). The upregulation of ISC signature genes was also observed in the *Lgr5-CreER;Bmpr1a*^*fl/fl*^ mice (Supplementary Fig. 4i).

### BMP does not interfere with Wnt signalling in Lgr5^+^ ISCs

**Figure 4:**
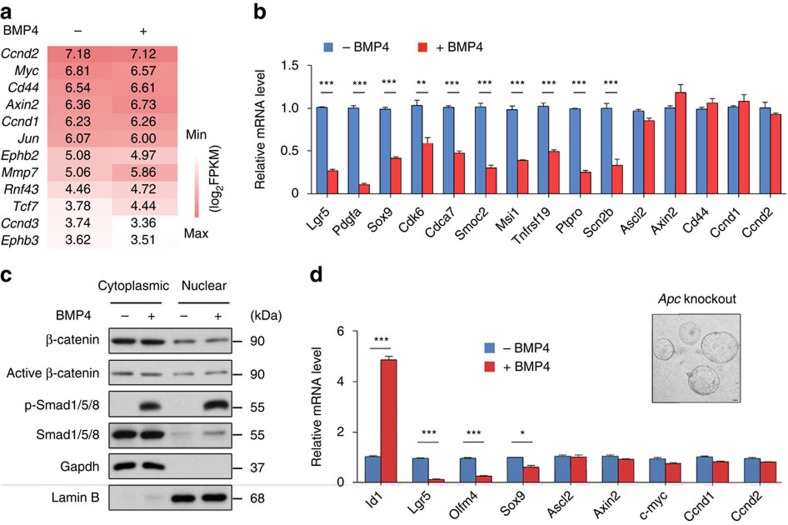
BMP activation has no direct inhibitory effects on Wnt signalling in Lgr5^+^ ISCs. (**a**) Heatmap showing the expression levels of several canonical Wnt target genes in Lgr5^+^ stem cells on BMP stimulation. (**b**) Quantitative RT–PCR verification of BMP-regulated genes and Wnt target genes in Lgr5^+^ stem cells. Data represent mean±s.e.m of *n*=3 independent experiments. ***P<0.001 by Student's *t*-tests. (**c**) Immunoblots of cytoplasmic and nuclear proteins from intestinal organoids treated with or without BMP4 for 4 h. Data are representative of three independent experiments. (**d**) Quantitative RT–PCR analysis of gene expression in *Apc* mutant organoids derived from intestinal tumours in *Vil-cre;Apc*^*wt/fl*^ mice that were treated with or without BMP for 4 h. Data represent mean±s.e.m of three independent experiments. *P<0.05, **P<0.01, ***P<0.001 by Student's *t*-tests. Scale bar, 50 μm.

It has been reported that BMP may repress intestinal renewal by suppressing Akt activity and thus blocking the nuclear accumulation of β-catenin in BrdU label-retaining quiescent stem cells^17^. However, our data indicate that BMP suppresses Lgr5^+^ ISC self-renewal through directly inhibiting stem cell signature genes rather than interfering with Wnt/β-catenin signalling. Consistent with this notion, the RNA-seq data also showed that 4 h treatment of BMP had no apparent effect on many classical Wnt target genes such as *Ccnd2*, *Axin2* and *Myc* (Fig. 4a). These results were also validated by quantitative reverse transcription–PCR (RT–PCR; Fig. 4b). To further assess whether BMP affects the nuclear translocation of β-catenin, we examined the subcellular distribution of β-catenin in the cultured intestinal organoids on BMP4 treatment for 4 h, the time point when BMP4 had an evident effect on the expression of stem cell signature genes. We found that BMP4 did not change the nuclear distribution of β-catenin although it enhanced the nuclear accumulation of p-Smad1/5/8 (Fig. 4c). Moreover, in the *Apc* KO organoids that display high Wnt activity due to the increased nuclear accumulation of β-catenin^40^ (Supplementary Fig. 1h), BMP was still able to suppress the expression of ISC signature genes without affecting Wnt targets (Fig. 4d). Taken together, our data suggest that BMP restricts the stemness of Lgr5^+^ ISCs through direct repression of ISC signature gene expression, but not through inhibition of Wnt/β-catenin signalling.

### Smad1 and Smad4 bind to the promoters of ISC signature genes

**Figure 5:**
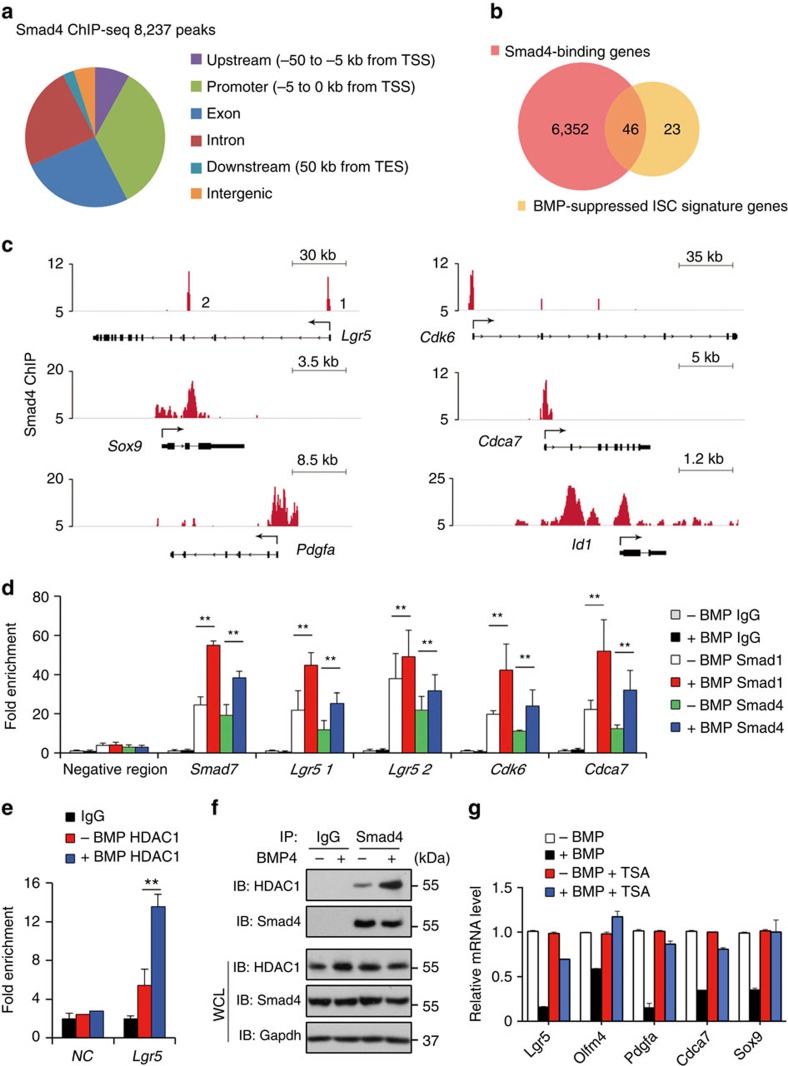
Smad proteins bind to the promoter regions of stem cell signature genes and recruit HDAC1 to repress transcription. (**a**) Genome-wide distribution of Smad4-binding sites in Lgr5^+^ stem cells. TES, transcription end site; TSS, transcription start site. (**b**) Venn diagram showing the overlap between BMP-downregulated signature genes with Smad4 bound genes. *P*=6.00E-12. (**c**) ChIP-seq signals for Smad4 binding at the representative genomic loci. *Id1* served as a positive control. (**d**) Lgr5^+^ stem cells were stimulated with or without BMP4 for 4 h and subjected to ChIP assays with anti-Smad1, anti-Smad4 antibodies or control IgG. Quantitative RT–PCR followed ChIP shows the enrichment of Smad1 or Smad4 in the promoter or enhancer regions of indicated genes. *Smad7* served as positive control. Data represent mean±s.e.m of three independent experiments. **P<0.01 by Student's *t*-test. (**e**) HDAC1 ChIP assays in Lgr5^+^ stem cells with or without 4 h BMP4 treatment. Data represent mean±s.e.m of three independent experiments. **P<0.01 by Student's *t*-test. (**f**) Intestinal organoids were treated with 10 ng ml^−1^ BMP4 for 4 h before being collected for immunoprecipitation (IP) with control IgG or anti-Smad4 antibody followed by western blotting. Protein expression was determined with whole-cell lysates (WCL). (**g**) Quantitative RT–PCR analysis of *Lgr5*, *Olfm4*, *Pdgfa*, *Cdca7* and *Sox9* in intestinal organoids after BMP stimulation in the presence or absence of 300 nM trichostatin A (TSA) for indicated time. For TSA treatment, the organoids were first pretreated with 300 nM TSA for 2.5 h. Data represent mean±s.e.m of three independent experiments.

Smad proteins are the central mediators of transforming growth factor-β/BMP signalling to regulate gene expression^8^,^9^,^10^,^11^,^13^. To explore whether BMP exerts its suppression of ISC signature genes through Smad proteins, we first performed Smad4 genome-wide chromatin immunoprecipitation and sequencing (ChIP-seq) analysis on Lgr5^+^ ISCs sorted from intestinal organoid cultured in normal condition. This is because the stem cells cultured in the normal medium containing 100 ng ml^−1^ Noggin still displayed a basal level of BMP activity (Supplementary Fig. 5a), probably due to accumulation of BMP ligands provided by Matrigel or secreted by organoids^32^,^41^. Consistent with this, self-renewal of Lgr5^+^ ISCs in organoids was significantly enhanced when supplemented with a higher concentration of Noggin (Supplementary Fig. 5b–d). ChIP-seq revealed 8,237 Smad4-binding sites in Lgr5^+^ ISCs, which were annotated to a total of 6,398 genes (Supplementary Data Set 2). Most of the Smad4-binding sites were located within 4 kb from the transcription start site, and of all binding sites, 34% were within the promoter regions, whereas 26% and 24% in exons and introns, respectively (Fig. 5a and Supplementary Fig. 6a). Consistently, a good correlation between BMP target genes bound by Smad4 and BMP function in Lgr5^+^ stem cells was observed (compare Supplementary Fig. 6b with Fig. 3d), and nearly 66% BMP-suppressed ISC signature genes overlapped with Smad4-occupied genes (Fig. 5b). For instance, *Lgr5*, *Sox9*, *Pdgfa*, *Cdk6* and *Cdca7* had Smad4 ChIP-seq signals at their genomic loci (Fig. 5c). We further verified the increased recruitment of both Smad1 and Smad4 to the promoter or enhancer regions of *Smad7*, *Lgr5*, *Cdk6* and *Cdca7* by ChIP–quantitative PCR in Lgr5^+^ ISCs after BMP stimulation for 4 h (Fig. 5d). However, we did not detect Smad4 binding in the promoter regions of the canonical Wnt targets, including *Axin2*, *EphB2* and *Cd44* in Smad4 ChIP-seq (data not shown), indicating that BMP selectively represses stem cell signature genes through Smad binding. On closer examination, we found that the Smad4-binding site around the transcription start site of Lgr5 is highly conserved across mammalian species and is close to the T-cell factor 4-binding site (Supplementary Fig. 6c), suggesting a possible convergence of BMP signalling and Wnt signalling in regulation of Lgr5 expression. However, as shown by ChIP assay, the occupancy of β-catenin to the Lgr5 promoter was not affected by Smad binding on BMP activation (Supplementary Fig. 6d).

To investigate how Smad binding represses the expression of stem cell signature genes, we further performed histone deacetylase 1 (HDAC1) ChIP assays in Lgr5^+^ stem cells. It has been shown that BMP-induced DNA binding of Smads can recruit the histone deacetylase HDAC1 and thus repress gene expression^42^,^43^. Indeed, BMP stimulated the binding of HDAC1 to the Lgr5 promoter (Fig. 5e). Moreover, BMP enhanced the interaction between Smad4 and HDAC1 in intestinal organoids (Fig. 5f), and trichostatin A, a potent inhibitor of HDACs, efficiently blocked the suppressive effects of BMP on stem cell signature gene expression (Fig. 5g). Together, the results strongly suggest that BMP/Smad signalling directly represses the expression of the stem cell signature genes in Lgr5^+^ stem cells and the repression is mediated by the Smad recruitment of HDAC1.

### Stem cell signature genes are more sensitive to BMP activity

**Figure 6:**
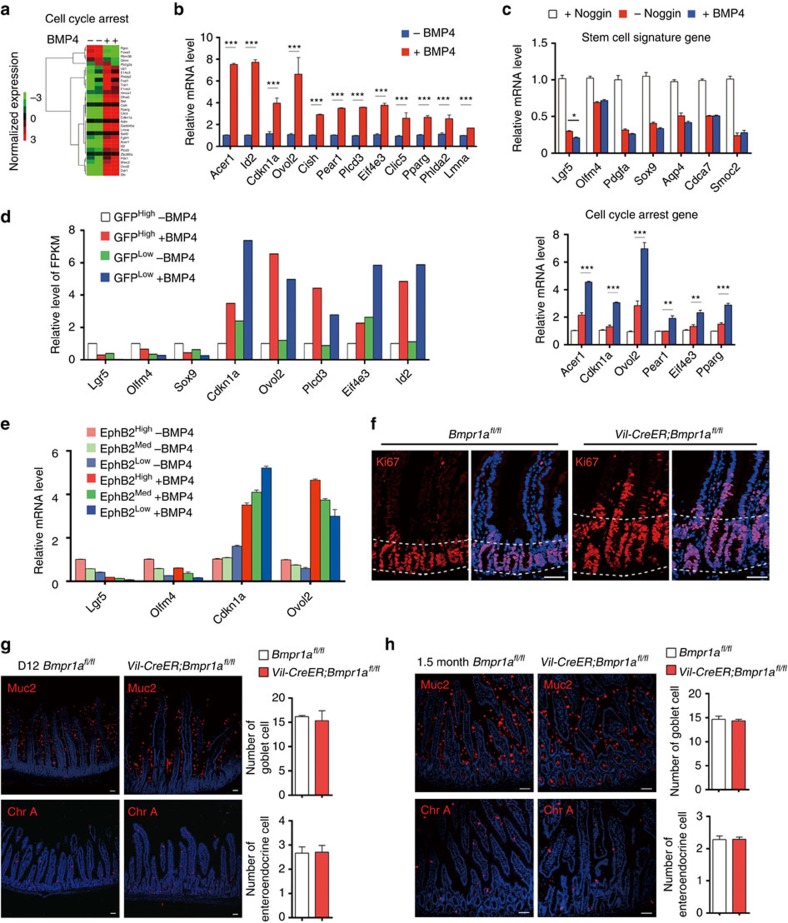
Low BMP activity restricts the self-renewal of Lgr5^+^ stem cells through modulating stem cell signature genes but not cell cycle arrest genes. (**a**) BMP-regulated genes in Lgr5^+^ stem cells after 4 h BMP stimulation were grouped into cell cycle arrest cluster. (**b**) The expression of the selected genes in the cell cycle arrest cluster were validated by quantitative RT–PCR in Lgr5^+^ stem cells sorted from intestinal organoids. Data represent mean±s.e.m of three independent experiments. **P<0.01, ***P<0.001 by Student's *t*-test. (**c**) The expression of the stem cell signature genes and cell cycle arrest genes were measured by quantitative RT–PCR in intestinal organoids from indicated groups after passaging. Data represent mean±s.e.m of three independent experiments. *P<0.05, **P<0.01, ***P<0.001 by Student's *t*-test. (**d**) Relative level of FPKM of indicated genes from Lgr5-GFP^high^ and Lgr5-GFP^low^ cells with or without BMP treatment for 4 h. (**e**) The expression of the stem cell signature genes and cell cycle arrest genes were measured by quantitative RT–PCR in EphB2^high^, EphB2^medium^ and EphB2^low^ cells sorted from intestinal organoids treated with or without BMP4 for 4 h. Data represent mean±s.e.m of three independent experiments. (**f**) Immunofluorescence staining of Ki67 in *Bmpr1a*^*fl/fl*^ and *Vil-CreER;Bmpr1a*^*fl/fl*^ mice at day 12. Dotted lines delineate the region of crypts. Nuclei were counter-stained with 4′,6-diamidino-2-phenylindole (DAPI). Images are representative of *n*=6 mice per genotype. (**g**,**h**) Muc2 immunofluorescence staining and chromogranin A immunofluorescence staining in intestine from *Vil-creER;Bmpr1a*^*fl/fl*^ and *Bmpr1a*^*fl/fl*^ mice at day 12 (**g**) and 1.5 month after induction (**h**). Nuclei were counter-stained with DAPI. Images are representative of *n*=6 mice per genotype. Quantifications of goblet cell and enteroendocrine cell number are shown aside. Data represent mean±s.e.m of *n*=5 mice per genotype. Scale bars, 50 μm.

Our RNA-seq analysis also revealed that BMP stimulated the expression of cell cycle arrest genes, such as *Cdkn1a* (p21), in Lgr5^+^ ISCs, which was further validated with quantitative RT–PCR (Fig. 6a,b). Detection of Smad4-binding signals at the genomic loci of representative genes suggested that they are direct targets of the BMP/Smad pathway (Supplementary Fig. 6e). Unlike the stem cell signature genes that were repressed by low BMP activity through Noggin withdrawal, the cell cycle arrest genes were only significantly induced on external addition of BMP4 in Lgr5^+^ stem cells (Fig. 6c), suggesting that stem cell signature genes are more sensitive to BMP activity. Thus, we proposed that low BMP activity specifically restricted the proliferation and stem cell potential of Lgr5^+^ ISC through modulation of stem cell signature genes but not cell cycle arrest genes (Figs 3a and 6c). On the other hand, high BMP can directly abolish the proliferation of not only Lgr5^+^ ISCs but also their proliferating progeny through inducing cell cycle arrest genes (Figs 3a and 6c). Indeed, BMP treatment led to the similar fold changes of cell cycle arrest genes such as *Cdkn1a* and *Ovol2* in both Lgr5-GFP^high^ (representing stem cells) and GFP^low^ (representing transient amplifying) cells^44^ (Fig. 6d). Similar results were obtained with EphB2-high, -medium and -low cells^45^ (Fig. 6e). Consistent with this, the proliferating transient amplifying cells in *Bmpr1a* cKO continually invaded into the villus where they normally stopped division as BMP activity was high in villus (Figs 1a and 6f). No apparent alterations of apoptosis process or differentiation of goblet and enteroendocrine lineages were detected in *Bmpr1a* mutant mice (Fig. 6g,h and data not shown), suggesting that BMP mainly functions in coordinating the self-renewal and proliferation.

### Smad proteins mediate the effects of BMP in Lgr5^+^ ISCs

**Figure 7:**
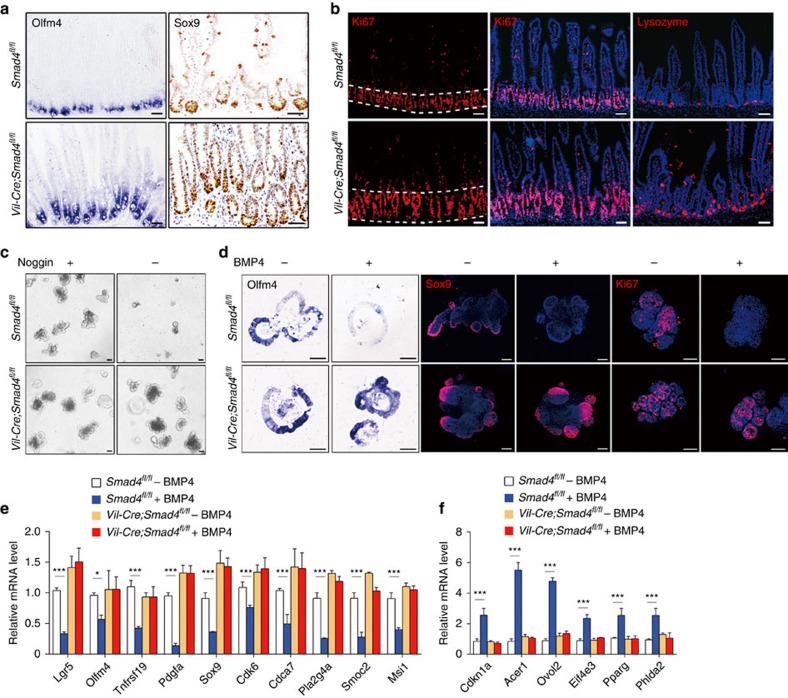
Smad4 mediates the negative regulation of BMP on Lgr5^+^ stem cell self-renewal. (**a**) *Olfm4 in situ* hybridization and Sox9 staining in intestines from 1-month-old *Smad4*^*fl/fl*^ and *Vil-Cre;Smad4*^*fl/fl*^ mice. Images are representative of *n*=5 mice per genotype. (**b**) Ki67 and lysozyme staining in intestines from 1-month-old *Smad4*^*fl/fl*^ and *Vil-Cre;Smad4*^*fl/fl*^ mice. Dotted lines delineate the region of crypts. Nuclei were counter-stained with 4′,6-diamidino-2-phenylindole (DAPI). Images are representative of *n*=5 mice per genotype. (**c**) Intestinal organoids derived from *Smad4*^*fl/fl*^ and *Smad4*^*KO*^ (*Vil-Cre;Smad4*^*fl/fl*^) mice were cultured in ENR (EGF, Noggin and R-spondin) or ER medium for 5 days and photographed. Images are representative of three independent experiments. (**d**) Intestinal organoids derived from *Smad4*^*fl/fl*^ and *Smad4*^*KO*^ (*Vil-Cre;Smad4*^*fl/fl*^) mice were treated with BMP4 for 36 h followed by *Olfm4 in situ* hybridization, Sox9 staining and Ki67 immunofluorescence staining. Nuclei were counter-stained with DAPI. Images are representative of three independent experiments. (**e**,**f**) *Smad4*^*fl/fl*^ and *Smad4*^*KO*^ (*Vil-Cre;Smad4*^*fl/fl*^) organoids were treated with BMP4 for 4 h and the mRNA level of indicated genes were measured by quantitative RT–PCR. Data represent mean±s.e.m of three independent experiments. *P<0.05, ***P<0.001 by Student's *t*-tests. Scale bars, 50 μm.

To directly address the role of Smad proteins in mediating BMP function in intestinal epithelium, we generated intestinal epithelium-specific *Smad4* KO mice by breeding *Smad4*^*fl/fl*^ mice with *Villin-Cre* (*Vil-Cre*) strain. Intestinal epithelium-specific loss of *Smad4* resulted in an obvious expansion of the Lgr5^+^ stem cells by 1 month after birth, as indicated by *Olfm4 in situ* hybridization and Sox9 immunostaining (Fig. 7a and Supplementary Fig. 7a). The mutant intestine also harboured significantly enlarged proliferative crypt compartment and increased number of Paneth cells (Fig. 7b and Supplementary Fig. 7b). As expected, the BMP-regulated stem cell signature genes displayed a significant increase in expression in the mutant crypts, while *Ascl2* remained unchanged (Supplementary Fig. 7c). All these phenotypes resemble those observed in *Bmpr1a* KO mice, supporting that Smad4 is a critical mediator of BMP signalling in intestinal epithelium.

To further explore the role of Smad4 in mediating the effects of BMP on Lgr5^+^ ISCs, we isolated crypts from the *Vil-Cre*;*Smad4*^*fl/fl*^ mice and their wild-type littermate. The wild-type crypts barely grew into organoids after 5-day withdrawal of Noggin in the culture medium, while Smad4 deletion entirely eliminated the requirement for Noggin (Fig. 7c). Consistent with this, the Smad4-deficient organoids retained the Lgr5^+^ stem cell population (Olfm4/Sox9-positive) and proliferative compartment (ki67-positive) even in the presence of BMP4, which is in contrast to the loss of these markers in wild-type organoids (Fig. 7d and Supplementary Fig. 7d). Importantly, at the transcription level, none of the BMP-regulated ISC signature genes and cell cycle-related genes responded to BMP on Smad4 ablation (Fig. 7e,f and Supplementary Fig. 7e,f). Taken together, these results confirm that BMP restricts the stemness of Lgr5^+^ stem cells through the Smad pathway.

## Discussion

BMP maintains intestine epithelium homeostasis by restricting its hyperproliferation. It was previously suggested that BMP might inhibit the intestinal proliferation by direct suppression of β-catenin nuclear translocation in BrdU-retaining cells^17^. However, the BrdU-retaining cells are quiescent stem cells and barely contribute to the intestinal daily renewal, whereas Lgr5^+^ ISCs actively mediate the fast renewal of intestinal epithelium and can fuel the growth of intestinal adenomas on *Apc* mutation^46^,^47^. Several *in vitro* studies indicate that BMP might inhibit Lgr5^+^ stem cell activity, but whether BMP directly regulates the self-renewal of Lgr5^+^ stem cells during the intestinal homeostasis is unknown^30^,^31^. It was recently suggested that GATA6 enables the self-renewal of Lgr5^+^ cancer stem cells by restricting BMP signalling, but how BMP exerts its functions and whether BMP restricts self-renewal of Lgr5^+^ stem cells in normal intestinal epithelium remain unclear^32^. Here we provide evidence for a critical role of epithelial BMP signalling in restricting Lgr5^+^ ISC self-renewal through direct repression of stem cell signature genes, highlighting that BMP limits the hyperproliferation of intestinal epithelium through constraining the Lgr5^+^ stem cell expansion under both homeostatic and injury conditions.

A recent study suggested that transgenic overexpression of the BMP antagonist Gremlin 1 in intestine resulted in intestinal polyposis with mixed morphology, resembling hereditary mixed polyposis syndrome^22^. This syndrome is mainly caused by mutations in *GREM* but not in other components in the canonical BMP pathway. The authors suggested that aberrant overexpression of Gremlin 1 in intestine initiates colonic tumorigenesis from villus cells but not from Lgr5^+^ stem cells. However, we observed the marked expansion of Lgr5^+^ stem cell compartment after loss of epithelial BMP activity, but failed to detect the existence of stem cell property in epithelial invaginations on the flank of villus in our model. Our findings are in consistent with a number of studies suggesting that tumour-initiating cells are derived from Lgr5^+^ stem cells^32^,^46^,^47^,^48^. As Gremlin 1 is also involved in several other signalling pathways such as VEGF, Slit/ROBO and Firillin^49^,^50^, it is possible that the phenotypes resulted from Gremlin overexpression may not be solely attributed to BMP inhibition. It remains controversial how important is epithelial BMP in intestinal polyp growth and cancer development. In the Noggin transgenic model^14^ and the Bmpr1a conditional KO model using interferon-inducible Mxl-cre^17^, intestinal polyps were frequently observed, but it is unknown whether or not mesenchymal BMP activity is important. Auclair *et al*.^51^ showed that epithelial BMP signalling is important for the terminal differentiation of the intestinal secretory cell lineage but not involved in *de novo* crypt formation and polyp growth using *Villin-Cre;Bmpr1a*^*fl/fl*^ mice. Interestingly, the *Villin-Cre;Bmpr1a*^*fl/fl*^ mice were embryonic lethal in our hands. The discrepancy could be due to mouse genetic background or efficiency of Bmpr1a deletion using different Cre lines. Therefore, we generated the inducible *Villin-CreER;Lgr5-CreER;Bmpr1a*^*fl/fl*^ and *Lgr5-CreER;Bmpr1a*^*fl/fl*^ strains to delete Bmpr1a in intestinal epithelium or specifically in Lgr5^+^ stem cells. Our data clearly show that epithelial BMP signalling plays an indispensable role in restricting Lgr5^+^ stem cell expansion and subsequent polyp growth. Another study suggests that epithelial Smad4 is dispensable for intestinal cancer development^52^, but the Cre lines they used may not specifically target intestinal epithelium cells^52^,^53^.

We observed that the enlarged Lgr5^+^ stem cell pool on Bmpr1a deletion significantly boosted the intestinal regeneration after ionizing radiation. It was hypothesized that irradiation damage causes Lgr5^+^ stem cell depletion, and ‘+4' quiescent stem cells marked by *Bmi1* or *Hopx* are responsible for the regeneration^5^,^27^. However, a number of studies indicate that the Lgr5^+^ cells also expresses these +4 markers, suggesting an overlap between +4 cells and Lgr5^+^ cells^5^,^38^,^54^. Moreover, Lgr5^+^ stem cells have been shown to be indispensable for the regeneration^35^,^55^. Our findings are in consistent with this: the enlarged Lgr5^+^ stem cell pool in *Bmpr1a* cKO mice facilitated the intestinal regeneration as there were more surviving Lgr5^+^ stem cells in the mutant mice.

By examining the expression of classical Wnt target genes and the nuclear β-catenin level, we did not observed the direct effect of BMP on Wnt signalling in Lgr5^+^ ISCs. However, as BMP downregulates a number of genes that mediate or promote Wnt signalling, such as *Lgr5*, we cannot exclude the possibility that BMP may inhibit Wnt activity in the long term. Nonetheless, our results are in accordance with the early observation showing that only rare adenomatous polyps at very late stage displayed high level of nuclear β-catenin while most benign polyps displayed normal level of β-catenin in nucleus in Noggin transgenic mice^14^. Although our results demonstrate that BMP functions through the Smad pathway but not through the Wnt/β-catenin pathway, our findings indicate the convergence of BMP and Wnt signalling at the transcriptional level to regulate the expression of stem cell signature genes and thereby Lgr5^+^ ISC self-renewal. In intestine, Lgr5^+^ ISCs reside in a specialized niche at the crypt base where Wnt signal is high and BMP signal is low, and the number of Lgr5^+^ ISCs remains largely constant in each crypts during homeostasis^5^,^56^. Our data showed that BMP acts as a faithful guard against the amplification of stem cell pool, thereby restricting the self-renewal ability of intestinal epithelium. However, the hyperactive Lgr5^+^ ISCs with impaired BMP signalling still gradually lose stem cell identity on moving away from the crypt base, which is probably due to the reduction of Wnt signals in the environment. This highlights the necessity of cooperation between BMP and Wnt signals in the maintenance of stem cell property under physiological conditions.

In contrast to the prevalent tumour formation on constitutive activation of Wnt signalling^57^, BMP inactivation usually causes benign polyps of the intestinal epithelium that confer high risk of colorectal cancer initiation^53^. Our results are in agreement with this observation. As a large proportion of the signature genes are direct targets of Wnt or Ascl2 that require high Wnt activity for expression^26^, BMP inactivation alone may not be enough to sustain the stem cell property in Wnt-low environment as it mainly exerts a negative control over the stem cell signature programme. However, those hyperproliferative Lgr5^+^ ISCs on BMP inactivation may readily undergo tumorigenesis on DNA damage or with gain-of-function mutations of Wnt/β-catenin signalling components. Indeed, the mice carrying both mutations in *Apc* and *Smad4* developed more malignant tumours, highlighting the role of BMP inactivation in tumour progression^58^.

In summary, our findings addressed the essential role of epithelial BMP signalling in restricting Lgr5^+^ stem cell expansion during intestinal homeostasis and injury and provide a mechanistic insight into the function of BMP in restricting Lgr5^+^ stem cell self-renewal. Our findings also suggest that this pathway or its downstream stem cell signature gene programme may be worthwhile therapeutic targets in colorectal cancer treatment.

## Methods

### Mice

*Vil-cre* mice^59^, *Lgr5-EGFP-IRES-creERT2*^1^ and *Apc*^*fl/fl*^ mice were obtained from Jackson Laboratory. *Bmpr1a*^*fl/fl*^ mice^60^ were kindly provided by Yuji Mishina, and *Vil-creER* mice^61^ were a gift from Sylvie Robine. *Smad4*^*fl/fl*^ mice^62^ were from Xiao Yang. All mice were back-crossed into the C57BL/6 genetic background for at least 10 generations. Both male and female mice ranging from 2 to 4 months old in age were used. No statistical method was used to predetermine sample size. Generally, we used at least five mice per genotype in each experiment. None of the animals was excluded from the experiment, and the animals used were not randomized. The investigators were not blinded to allocation during experiments and outcome assessment. For Cre induction, mice were intraperitoneally injected with 100 μl tamoxifen in sunflower oil at 20 mg ml^−1^ for 5 consecutive days. For BrdU pulse-chase experiment, each mouse with indicated genotypes was injected with a single BrdU injection (1 mg) and analysed after different time. For irradiation, mice received a single dose of abdominal X-ray radiation (10 or 14 Gy) and were then analysed at different time points. For *in vivo* lineage tracing, *Lgr5-CreER;td-Tomato* and *Lgr5-CreER;Bmpr1a*^*fl/fl*^*;td-Tomato* mice were intraperitoneally injected once with 50 μl tamoxifen in sunflower oil at 20 mg ml^−1^ and analysed after 3.5 days. All animal studies were performed in accordance with the relevant guidelines and under the approval of the Institutional Animal Care and Use Committee of our institute.

### Isolation of intestinal crypts and organoid culture

Intestinal crypts were isolated and cultured as previously described^63^. Briefly, mouse intestine was cut longitudinally and washed three times with cold PBS. Villi were carefully scraped away and small pieces (5 mm) of intestine were incubated in 2 mM EDTA in PBS for 40 min on ice. These pieces were then vigorously suspended in cold PBS and the mixture was passed through 70 μm cell strainer (BD Biosciences). The crypt fraction was enriched through centrifugation (3 min at 300–400*g*). Then the crypts were embedded in Matrigel (BD Biosciences) and seeded on 48- or 24-well plate. After polymerization, crypt culture ENR medium (Advanced DMEM/F12 supplemented with Penicillin/Streptomycin, GlutaMAX-I, N2, B27 and *N*-acetylcysteine (Sigma-Aldrich)) containing EGF (50 ng ml^−1^, Invitrogen), Noggin (100 ng ml^−1^, R&D) and R-spondin1 (500 ng ml^−1^, R&D) was added and refreshed every 2 days. For BMP4 treatment, organoids were cultured in ENR medium for 2.5 days followed by cultured in ENR medium or ERB (EGF, R-spondin1 and 10 ng ml^−1^ BMP4; R&D) medium for indicated time. For Noggin withdrawal, organoids were cultured in ENR medium for 2.5 days followed by passaging. ENR medium or ER medium was added for indicated time, respectively. For passaging, the organoids embedded in Matrigel in each well were directly suspended in 1 ml cold PBS after removal of medium and were pelleted by centrifugation (3 min at 300–400*g*). The pelleted organoids were embedded in fresh Matrigel and seeded on plate followed by addition of culture medium as indicated in the figure legends. For *in vitro* lineage tracing, organoids derived from *Lgr5-CreERT2;td-Tomato* mice were stimulated by 4-OHT (30 nM) for 12 h followed by passaging.

### RNA-seq

Organoids derived from *Lgr5-EGFP-IRES-creERT2* mice were cultured in ENR medium for 2.5 days. The medium was removed and the Matrigel containing organoids were washed one time gently with warm PBS. Fresh ENR medium or ERB medium was then added for 4 h. The organoids were dissociated into single-cell suspension. Single Lgr5-GFP^high^ cells were then sorted by flow cytometry. RNA was purified from 100,000 single Lgr5-GFP^high^ cells using the RNeasy Mini Kit (Qiagen) and converted into cDNA libraries using the Ovation RNA-Seq System V2 kit (NuGEN). High-throughput sequencing was performed using the Illumina HiSeq 2000. The RNA-seq was carried out with two biological replicates. RNA-seq data were uniquely mapped to mm10 genome by TopHat v1.4.1 (ref. 64). Expression values were assigned to gene level by Cufflinks v1.3.0 (ref. 65). Genes with absolute log_2_-transformed fold changes ≥0.6 were regarded as differentially expressed genes, and a threshold of *q* value <0.05 was used. A list of BMP target genes from RNA-seq data is presented in Supplementary Data Set 1. Hierarchical clustering of gene fold changes was generated by Cluster 3.0 (http://bonsai.hgc.jp/~mdehoon/software/cluster/software.htm) and visualized by JavaTreeView. Gene set enrichment analyses (GSEA) were performed with the GSEA platform of the Broad Institute (http://www.broadinstitute.org/gsea/index.jsp) using the pre-ranked lists of gene fold changes against public gene sets of intestinal gene signature. GO analysis was performed with Hypergeometric test by using differentially expressed genes against gene sets from GO database (http://geneontology.org/) and KEGG pathway database (http://www.genome.jp/kegg/pathway.html).

### ChIP and ChIP-seq

All ChIP assays were performed in Lgr5^+^ stem cells sorted from cultured organoids. In brief, organoids derived from *Lgr5-EGFP-IRES-creERT2* mice were cultured in ENR medium for 2.5 days. For certain experiments, the organoids were stimulated with BMP4 (10 ng ml^−1^) for 4 h. One million single Lgr5^+^ stem cells were obtained in each group through flow cytometry as described above. The cells were crosslinked with 1% formaldehyde for 8 min at room temperature, resuspended in lysis buffer and collected in shearing buffer according to the manufacturer's instructions (iDeal ChIP-seq kit, Diagenode). The chromatin was sheared on a Covaris S2 system (Covaris) with the following settings: shearing time 20 min; 2% duty cycle; intensity 3; and cycle per burst 200. ChIP assays were then carried out with the manufacturer's instructions, using anti-Smad4 (ref. 66) or anti-Smad1 (CST, 6944, 1:2,000) antibodies. For ChIP–quantitative PCR, the immunoprecipitated DNA or input DNA was analysed using quantitative real-time PCR with a LightCycler 480 system. All the primers used were available in Supplementary Data Set 3. For ChIP-seq, Smad4 immunoprecipitated DNA was used for preparation of indexed libraries with MicroPlex Library Preparation kit (Diagenode), and sequenced on an Illumina HiSeq 2000. ChIP-seq data were uniquely mapped to mm10 genome by Bowtie v1.1.1, and peaks detection was done by MACS with default cutoff. Peaks were assigned to the nearest genes using Homer (http://homer.salk.edu/homer/index.html). Heatmaps of Smad4 enrichment across 8 kb regions were visualized using JavaTreeView.

### Flow cytometry

Fresh intestinal crypts or cultured organoids derived from *Lgr5-EGFP-IRES-creERT2* mice were incubated in TrypLE (Invitrogen) for 20 min at 37 °C to obtain single-cell suspension. For cultured organoids, the organoids embedded in Matrigel were first suspended in cold PBS after removal of medium and were pelleted by centrifugation (3 min at 300–400*g*). The dissociated cells were passed through 40 μm cell strainer (BD) and single Lgr5-EGFP^high^ cells were sorted by flow cytometry (MoFlo XDP, Beckman). For EphB2 staining, the dissociated cells were first incubated in primary antibody (R&D, AF467) in 3% BSA for 30 min, followed by wash with PBS. The cells were then incubated in secondary antibody in 3% BSA for another 30 min. Then the cells were washed twice and subjected to the FACS analysis. The acquired data were analysed with Summit 5.3 software.

### Quantitative RT–PCR

Total RNA was extracted with RNeasy Mini Kit (Qiagen) and cDNA was prepared using Revertra Ace (Toyobo). Real-time PCR reactions were performed in triplicates on a LightCycler 480 (Roche). Primers used were listed in Supplementary Data Set 3.

### Immunofluorescence and immunohistochemistry

For immunofluorescence, intestine isolated from mice was washed with cold PBS for several time, fixed with 4% formaldehyde solution and embedded in 4% low-melting-point agarose (Invitrogen) as described previously^67^. The sections were prepared with vibrating blade microtome (HM650, Microm) and permeabilized with PBDT solution (1% BSA, 1% dimethylsulfoxide and 2% Triton X-100 in PBS) for 1 h at room temperature. For organoid staining, isolated organoids were first fixed for 1 h with 4% paraformaldehyde at room temperature, permeabilized for 20 min with 0.2% Triton X-100 on ice and blocked in 5% BSA for 1 h at room temperature. The sections or organoids were then incubated overnight with the primary antibody at 4 °C. The following primary antibodies were used: rabbit anti-Ki67 (Abcam, ab15580, 1:100); rabbit anti-p-Smad1/5/8 (CST, 9511, 1:100); rabbit anti-lysozyme (Dako, F0372, 1:200); rabbit anti-Muc2 (Santa Cruz, sc-15334, 1:300); goat anti-ChromograninA (Santa Cruz, sc-1488, 1:300); and mouse anti-β-catenin (Sata Cruz, sc-7963, 1:300). The fluorescein-labelled secondary antibodies (Life Technologies, 1:300) were applied for 1 h at room temperature. TUNEL assay was performed using the *in situ* cell death detection kit (Roche). Confocal laser scanning was done on a Zeiss LSM 710 NLO Laser Scanning Microscope or an Olympus FV1200 Laser Scanning Microscope. For immunohistochemistry, formalin-fixed, paraffin-embedded intestine sections (5 μm) were de-paraffinized in isopropanol and graded alcohols, followed by antigen retrieval, and endogenous peroxidase quenched by H_2_O_2_. Sections were then blocked with 5% serum for 30 min and incubated overnight with rabbit anti-Sox9 (Millipore, AB5535, 1:200) at 4 °C. Secondary horseradish peroxidase-conjugated anti-rabbit antibody (Invitrogen, 1:200) was added for 2 h followed by development with DAB+ chromogen according to the manufacturer's (Dako Cytomation) recommendations. Slides were counterstained with Mayer's haematoxylin, dehydrated and mounted with Eukitt.

### *In situ* hybridization

Intestines from mice were flushed with cold PBS and fixed overnight in formalin. Samples were then dehydrated and embedded in paraffin, sectioned at 5 μm and processed to *in situ* hybridization. The sections were de-waxed, rehydrated, pretreated and hybridized overnight for 12 h at 65 °C with probes. After several times of wash, sections were incubated in blocking solution for 2 h followed by incubation with alkaline phosphatase-conjugated anti-digoxigenin (1:2,000; Roche) overnight at 4 °C. The sections were washed and further incubated with AP substrate (Roche) for appropriate time depending on signal strength. *Olfm4* fragment (2–1622) and *Pdgfa* fragment (313–709) were cloned into EZ-T vector. Probes were then generated through *in vitro* transcription with Roche *in vitro* transcription kit.

### Immunoblotting and immunoprecipitation

Protein lysates were prepared from intestinal epithelium or intestinal organoids. These assay were performed as previously described^68^. The following primary antibodies were used: rabbit anti-Smad1 (CST, 6944); rabbit anti-p-Smad1/5/8 (CST, 9511); rabbit anti-HDAC1 (Abcam, ab7028); mouse anti-active β-catenin (Millipore, 05–665); rabbit anti-Smad4 (Santa Cruz, sc-7154); and mouse anti-tubulin (Santa Cruz, sc-8035). Original immunoblots are shown in Supplementary Fig. 8.

### Statistics

All experiments were carried out with at least three biological replicates. Data shown in column graphs represent the mean±s.e.m., as indicated in the figure legends. When normality could be assumed, Student's *t*-tests was used to compare difference between two groups, otherwise the Mann–Whitney test was chosen. *P<0.05, **P<0.01, ***P<0.001. Statistical analysis was performed with GraphPad Prism6 software. For western blotting, immunohistochemistry, *in situ* hybridization and immunofluorescence, representative images are shown. Each of these experiments was independently repeated at least three times. No statistical method was used to predetermine sample size, and the experiments were not randomized. The investigators were not blinded to allocation during experiments and outcome assessment.

### Data availability

The authors declare that all data supporting the findings of this study are available within the article and its Supplementary Information Files.

## Additional information

**How to cite this article:** Qi, Z. *et al*. BMP restricts stemness of intestinal Lgr5^+^ stem cells by directly suppressing their signature genes. *Nat. Commun.* **8,** 13824 doi: 10.1038/ncomms13824 (2017).

**Publisher's note:** Springer Nature remains neutral with regard to jurisdictional claims in published maps and institutional affiliations.

## Supplementary Material

Supplementary Information Supplementary Figures

Supplementary Dataset 1 BMP target genes in Lgr5+ stem cells

Supplementary Dataset 2 Smad4 binding genes in Lgr5+ ISCs

Supplementary Dataset 3 PCR primers
