# Supplementary material for: BMP restricts stemness of intestinal Lgr5+ stem cells by directly suppressing their signature genes
Source: Nat Commun. 2017 Jan 6;8:13824. doi: 10.1038/ncomms13824 (PMC5227110; doi:10.1038/ncomms13824)
Supplement: Supplementary Information — Supplementary Figures [file ncomms13824-s1.pdf]

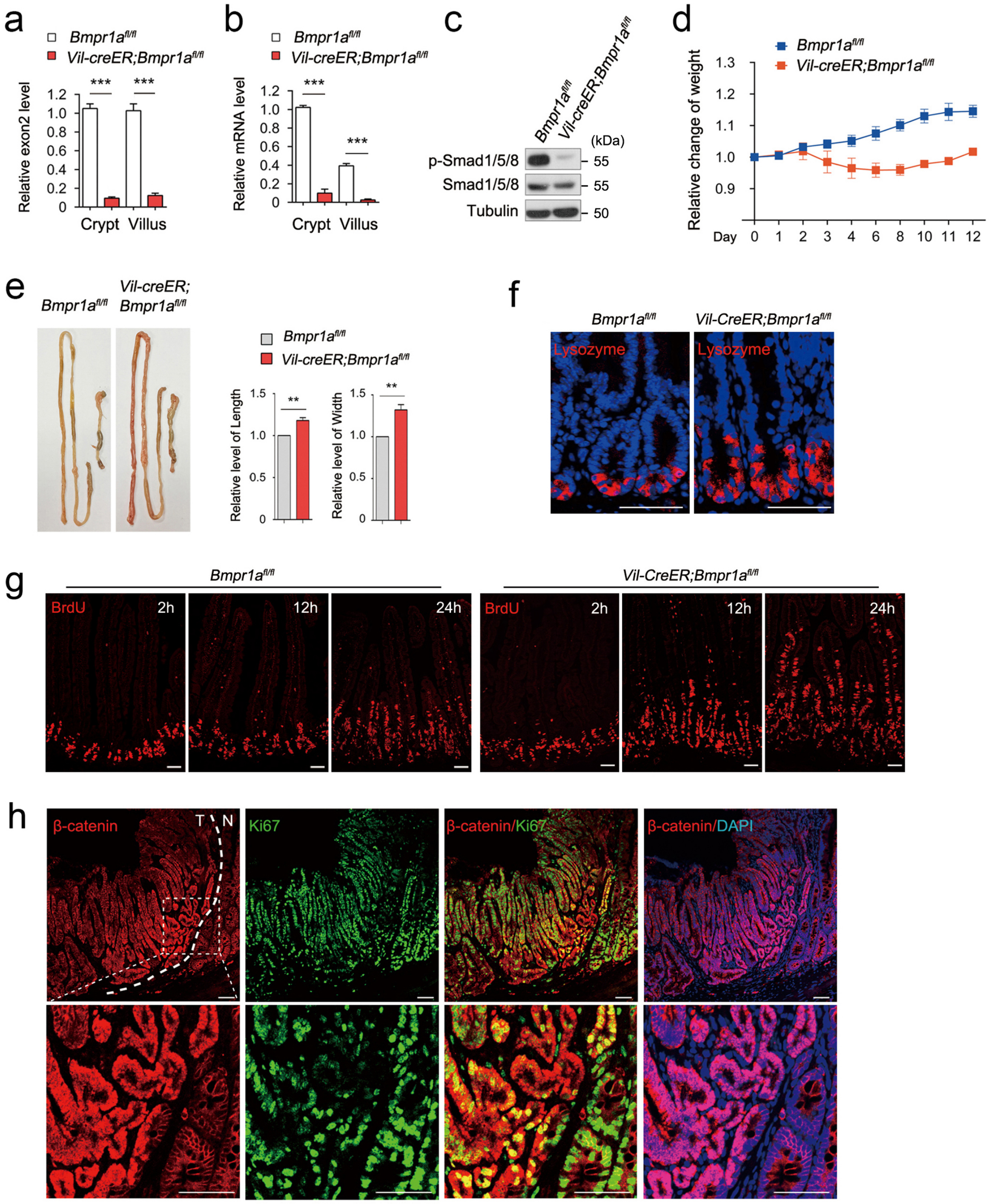

Supplementary Figure 1 BMP restricts Lgr5^{+} ISC self-renewal during homeostasis through a Wnt/β-catenin-independent pathway.

(**a**) Quantitative genomic PCR of crypt or villus DNA from *Bmpr1a*$^{fl/fl}$ and *Vil-creER;Bmpr1a*$^{fl/fl}$ mice at day 12 after the first injection of tamoxifen. Data represent mean ± s.e.m of n=6 mice. ****P* < 0.001 by Student's *t*-tests. (**b**) Quantitative RT-PCR of *Bmpr1a* expression in crypt or villus from the mice with indicated genotypes at day 12. Data represent mean ± s.e.m of n=6 mice. ****P* < 0.001 by Student's *t*-tests. (c) Immunoblots of small intestine epithelium from *Bmpr1a*$^{fl/fl}$ and *Vil-creER;Bmpr1a*$^{fl/fl}$ mice

at day 12. Data are representative of three independent experiments. (d) Body weight curve of *Bmpr1a*$^{fl/fl}$ and *Vil-creER;Bmpr1a*$^{fl/fl}$ mice before analysis. (**e**) Macroscopic images of intestines from *Bmpr1a*$^{fl/fl}$ and *Vil-creER;Bmpr1a*$^{fl/fl}$ mice at day 12 and quantification of the intestine length and width as indicated. Images are representative of n=6 mice per genotype. Data represent mean ± s.e.m of n=6 mice per genotype. ***P < 0.001 by Student's *t*-tests. (**f**) Immunofluorescence staining of Lysozyme in *Vil-CreER;Bmpr1a*$^{fl/fl}$ and *Bmpr1a*$^{fl/fl}$ mice at day 12. Nuclei were counter-stained with DAPI. Images are representative of n=6 mice per genotype. (**g**) Immunofluorescence staining for BrdU incorporation 2, 12 or 24 h after a single BrdU pulse in *Vil-CreER;Bmpr1a*$^{fl/fl}$ and *Bmpr1a*$^{fl/fl}$ mice. BrdU was injected at day 12. Images are representative of n=5 mice per genotype. (**h**) β-catenin and Ki67 immunofluorescence staining in transformed crypts compared with neighboring normal ones at a representative dysplastic region in intestine from 5 month-old *Vil-Cre;Apc*$^{wt/fl}$ mice. The lower panels show the enlargements of boxed area. T stands for tumor, and N stands for normal tissue. Nuclei were counter-stained with DAPI. Images are representative of n=4 mice per genotype. Scale bars, 50 μm.

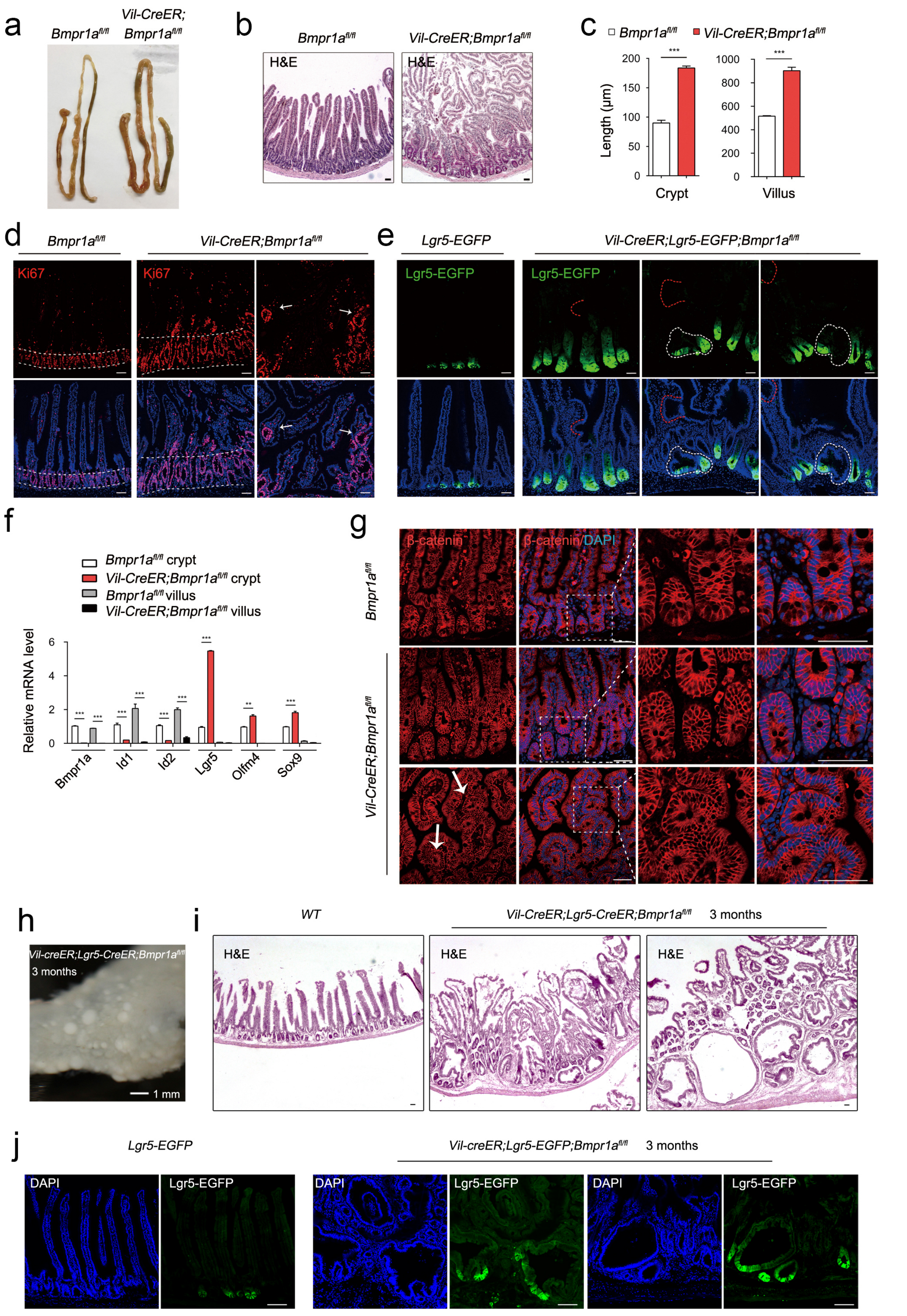

Supplementary Figure 2 Loss of BMP in intestinal epithelium drives intestinal dysplasia and polyp formation.

(**a**) Macroscopic images of intestines from *Bmpr1a*$^{fl/fl}$ and *Vil-creER;Bmpr1a*$^{fl/fl}$ mice at 1.5 months after 5-day tamoxifen administration. Images are representative of n=5 mice per genotype. (**b,c**) H&E staining of proximal jejunum sections and quantification of crypt length and villus length in *Bmpr1a*$^{fl/fl}$ and *Vil-creER;Bmpr1a*$^{fl/fl}$ mice. Images are representative of n=5 mice per genotype. Data represent mean ± s.e.m of n=5 mice per genotype. ***P < 0.001 by Student's *t*-tests. (**d**) Proximal jejunum sections were stained with Ki67. Dotted lines demarcate crypt region. Arrows mark crypt-like epithelium invaginations containing Ki67+ cells on the flanks of villus. Nuclei were counter-stained with DAPI**.** Images are representative of n=5 mice per genotype. (**e**) Lgr5-EGFP fluorescence of proximal jejunum sections in *Bmpr1a*$^{fl/fl}$ and *Vil-creER;Bmpr1a*$^{fl/fl}$ mice. Dotted red lines demarcate villus invaginations, while dotted white lines indicate the microadenoma-like structures. Nuclei were counter-stained with DAPI. Images are representative of n=5 mice per genotype. (**f**) Quantitative RT-PCR analysis of *Bmpr1a*, *Id1*, *Id2*, *Lgr5*, *Olfm4* and *Sox9* expression in crypt or villus from *Bmpr1a*$^{fl/fl}$ and *Vil-creER;Bmpr1a*$^{fl/fl}$ mice. Data represent mean ± s.e.m of three independent experiments. **P < 0.01, ***P < 0.001 by Student's *t*-tests. (**g**) β-catenin immunofluorescence staining in intestine from *Bmpr1a*$^{fl/fl}$ and *Vil-creER;Bmpr1a*$^{fl/fl}$ mice**.** Arrows mark crypt-like epithelium invaginations. Nuclei were counter-stained with DAPI. Images are representative of n=5 mice per genotype. (**h**) Polyps in duodenum from *Vil-CreER;Lgr5-CreER;Bmpr1a*$^{fl/fl}$ mice 3 months after 5-day tamoxifen injections. (**i**) H&E staining of polyps in the mutant mice. (**j**) Representative images of the Lgr5-EGFP^{+} stem cells at the base of polyps. Scale bars, 100 μm. Scale bars, 50 μm.

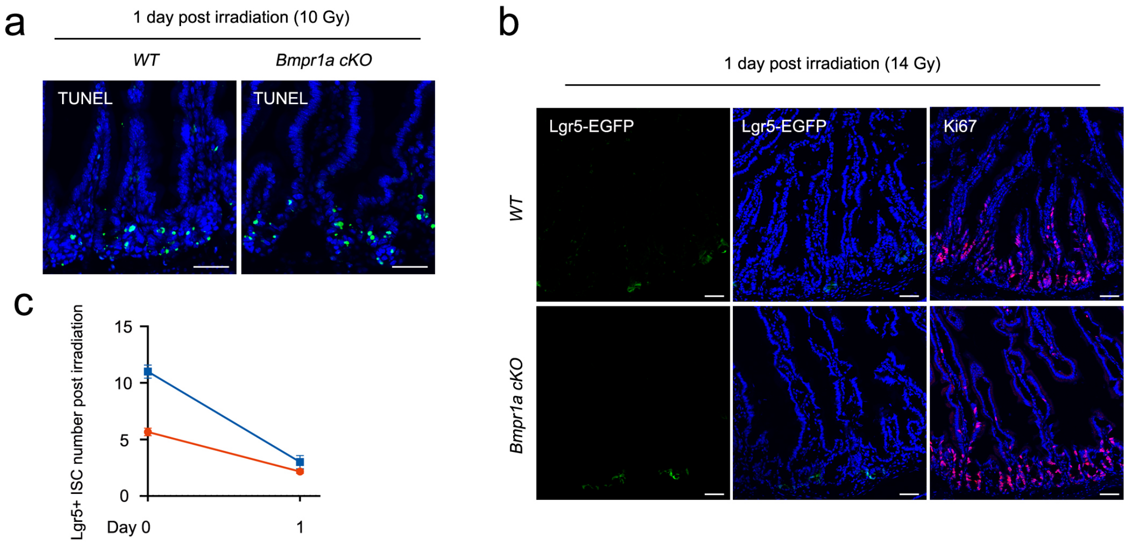

Supplementary Figure 3 Lgr5^{+} stem cells in *Bmpr1a cKO* mice do not show radioresistance.

(**a**) Representative images of TUNEL assays in jejunum sections from *Bmpr1a*$^{fl/fl}$ and *Vil-CreER;Bmpr1a*$^{fl/fl}$ mice at day 1 after 10 Gy abdominal X-ray radiation (n=5 mice per genotype). (**b**) Representative images of Lgr5-EGFP immunofluorescence and Ki67 staining in jejunum sections from *Bmpr1a*$^{fl/fl}$ and *Vil-CreER;Bmpr1a*$^{fl/fl}$ mice at different time points after 14 Gy abdominal X-ray radiation (n=5 mice per genotype). The mice were irradiated two weeks following 5-day tamoxifen induction. (**c**) Quantification of Lgr5^{+} stem cell number in *Bmpr1a*$^{fl/fl}$ and *Vil-CreER;Bmpr1a*$^{fl/fl}$ mice at different time points after 14 Gy abdominal X-ray radiation. Data represent mean ± standard error of the mean (s.e.m.) of n=5 mice per genotype. Scale bars, 50 μm.

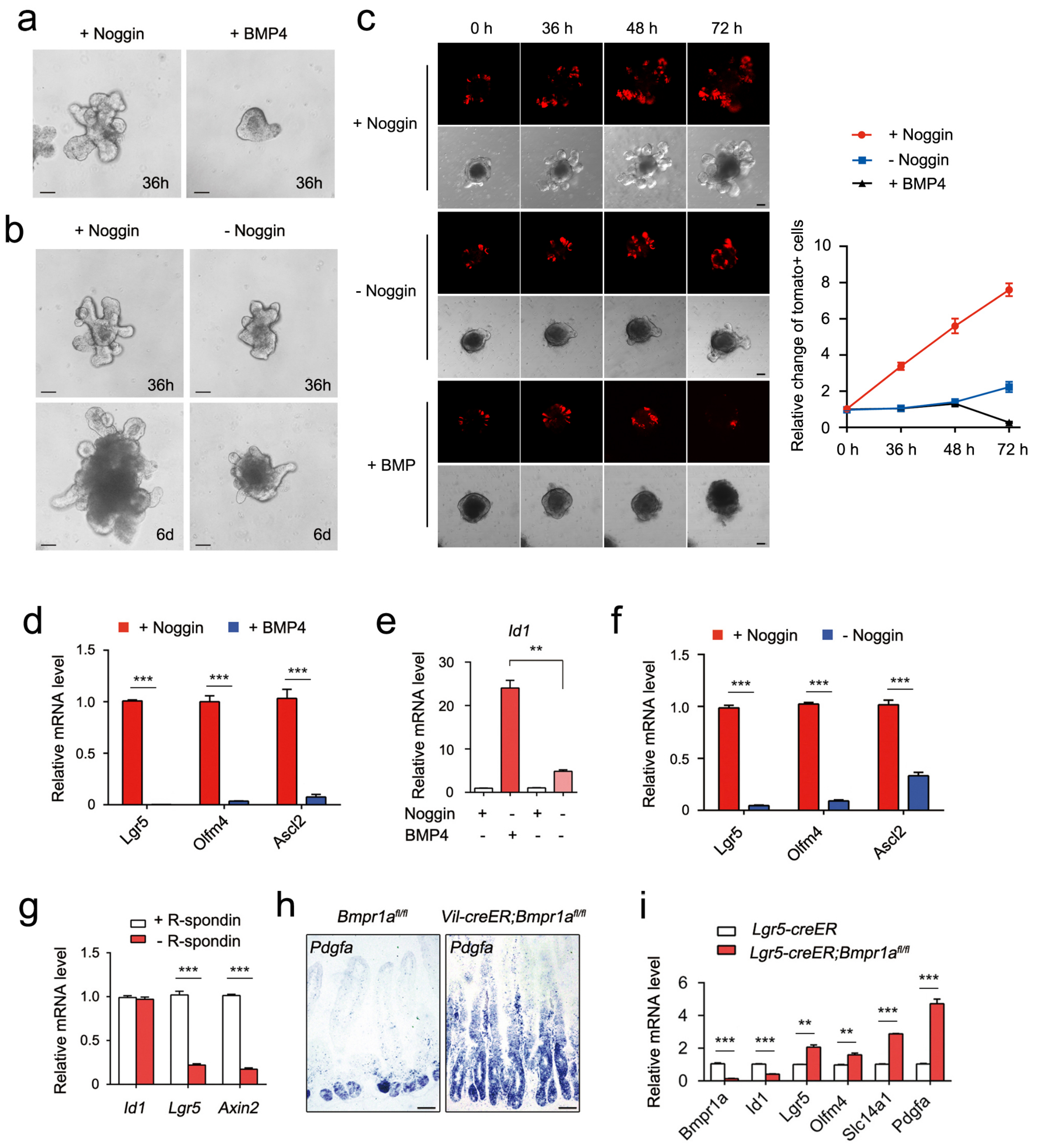

Supplementary Figure 4 BMP directly represses proliferation and expression of known stem cell markers of intestinal organoids *in vitro*.

(**a**) The intestinal crypts were cultured to form organoids for 2.5 days in the ENR (EGF, Noggin and R-spondin) medium followed by direct BMP4 treatment for 36 hours. Images are representative of three independent experiments. (**b**) The intestinal crypts were cultured to form organoids for 2.5 days in the ENR (EGF, Noggin and R-spondin) medium followed by passaging. Images are representative of three independent experiments. (**c**) Lineage tracing of $Lgr5^+$ stem cells in organoids derived from *Lgr5-CreERT2;td-Tomato* mice in indicated groups. 2-day old organoids were stimulated by 4-OHT for 12 hours followed by passaging. Representative images of tomato immunofluorescence in each groups at different time points were shown (n=3 independent experiments).

Quantification of tomato+ cells were shown aside. (**d**) Quantitative RT-PCR analysis of *Lgr5*, *Olfm4* and *Ascl2* in intestinal organoids after BMP stimulation for 24 hours. Data represent mean ± s.e.m of three independent experiments. ***P < 0.001 by Student's *t*-tests. (**e**) Quantitative RT-PCR analysis of *Id1* in intestinal organoids after BMP stimulation or Noggin withdrawal for 4 hours. Data represent mean ± s.e.m of three independent experiments. **P < 0.01 by Student's *t*-tests. (**f**) Quantitative RT-PCR analysis of *Lgr5*, *Olfm4* and *Ascl2* in intestinal organoids after Noggin withdrawal for 24 hours. Data represent mean ± s.e.m of three independent experiments. ***P < 0.001 by Student's *t*-tests. (**g**) Quantitative RT-PCR analysis of *Id1*, *Lgr5* and *Axin2* expression in organoids cultured in ENR medium or EN medium for 4 hours after passaging. Data represent mean ± s.e.m of three independent experiments. ***P < 0.001 by Student's *t*-tests. (**h**) In situ hybridization of *Pdgfa* in intestines from $Bmpr1a^{fl/fl}$ and $Vil\text{-}creER;Bmpr1a^{fl/fl}$ mice at day 12. Images are representative of three independent experiments. (**i**) Quantitative RT-PCR analysis of *Bmpr1a*, *Id1*, *Lgr5*, *Olfm4*, *Slc14a1* and *Pdgfa* expression in $Lgr5^+$ stem cells sorted from mice with indicated genotypes at day 12. Data represent mean ± s.e.m of n=4 mice per genotype. **P < 0.01, ***P < 0.001 by Student's *t*-tests. Scale bar, 50 µm.

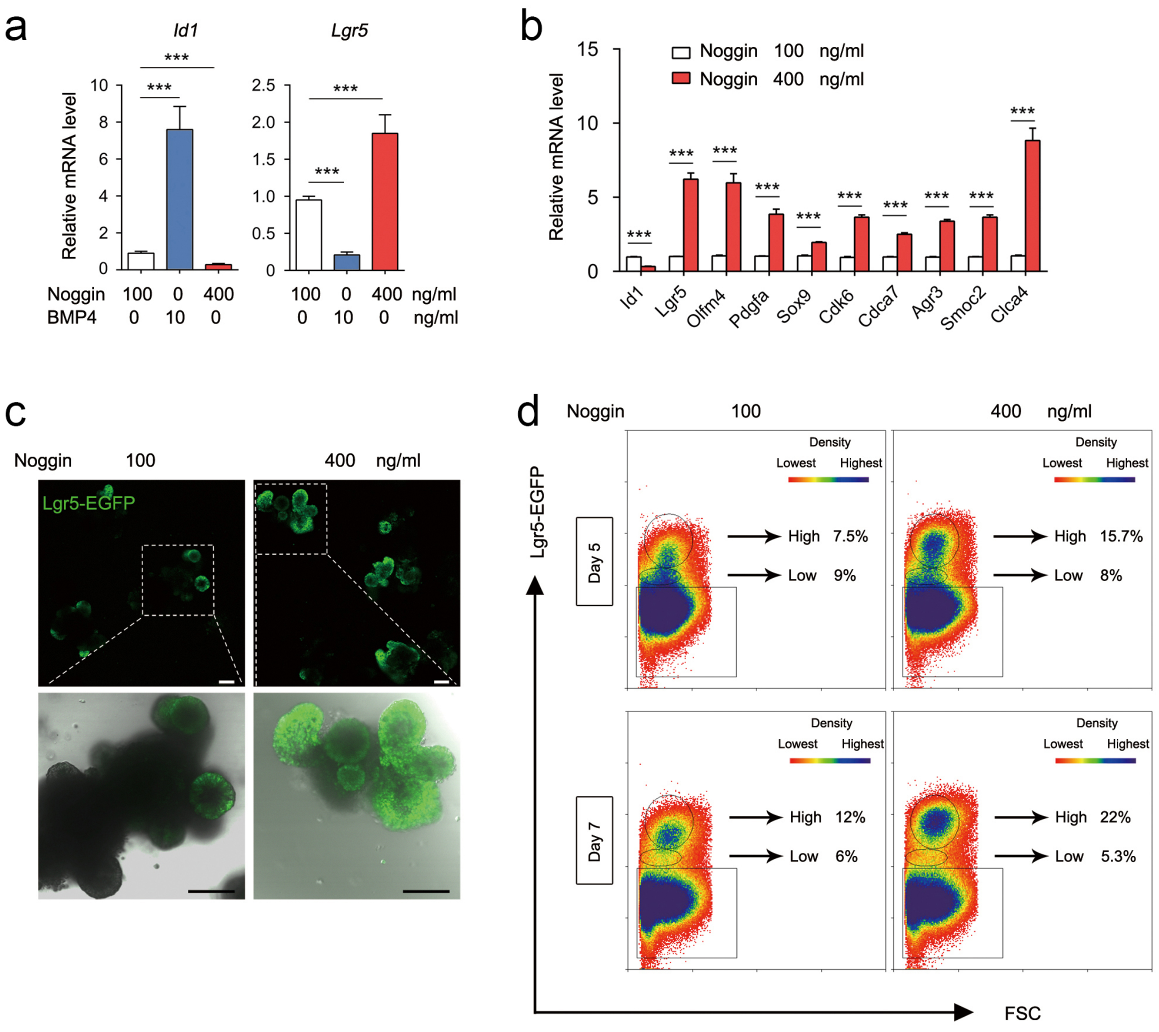

Supplementary Figure 5 Basal BMP activity constrains Lgr5^{+} stem cell expansion in the organoids cultured in the normal ENR medium.

(**a**) Increase in Noggin concentration (100 ng ml^{-1} or 400 ng ml^{-1}) significantly blocked basal BMP activity, as indicated by downregulation of *Id1* and upregulation of *Lgr5* in organoids. Data represent mean ± s.e.m of three independent experiments. ***$P <$ 0.001 by Student's *t*-tests. (**b**) Quantitative RT-PCR analysis of *Id1* and signature gene expression in organoids cultured for 5 days in the presence of Noggin. Data represent mean ± s.e.m of three independent experiments. ***$P < 0.001$ by Student's *t*-tests. (**c**) Confocal and bright-field images of Lgr5-EGFP (for Lgr5^{+} stem cells) in organoids cultured for 7 days with different amount of Noggin. Images are representative of three independent experiments. (**d**) Flow cytometry analysis of Lgr5-EGFP expression 5 days or 7 days after cultured with different amount of Noggin. Images are representative of three independent experiments. Scale bar, 50 μm.

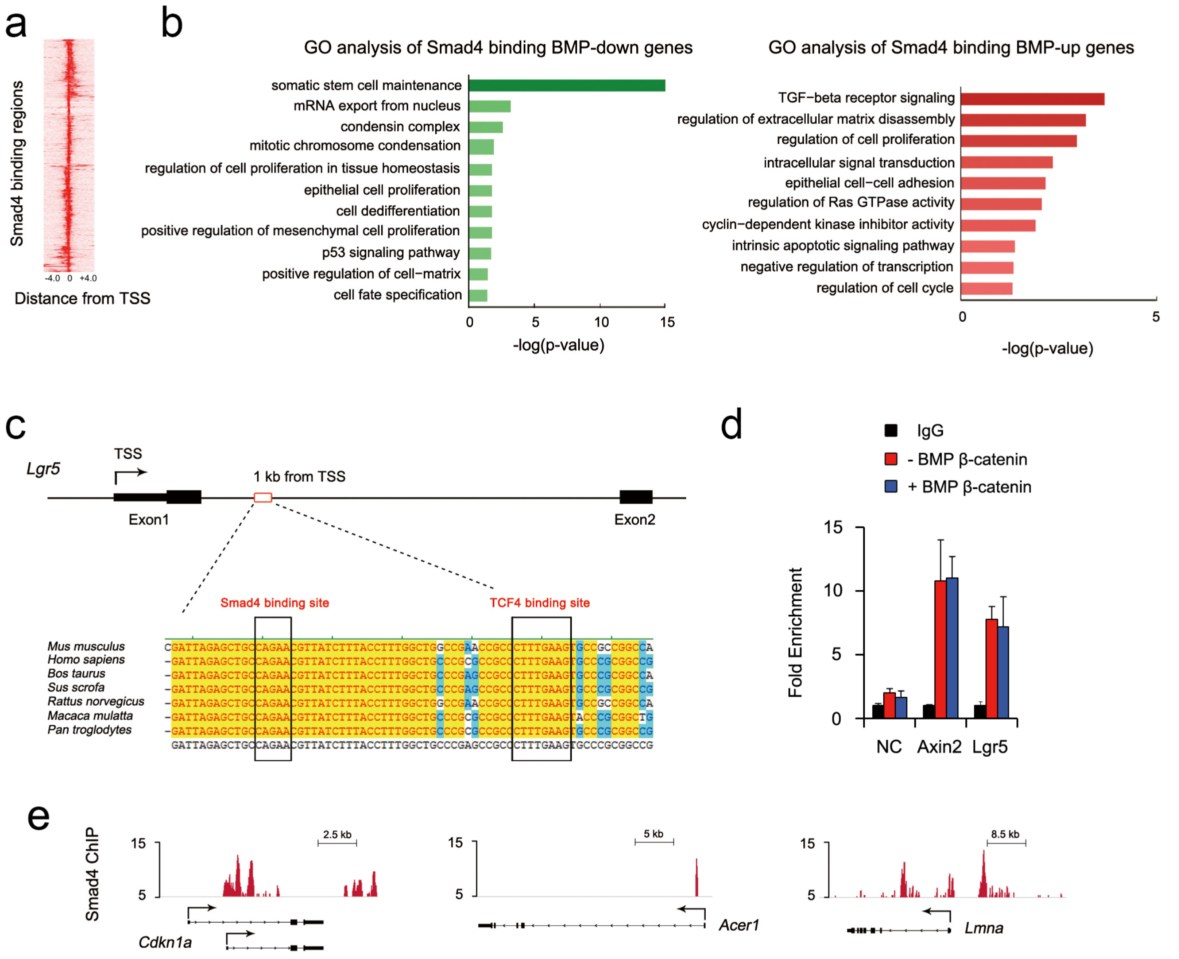

Supplementary Figure 6 Smad4 binds to the promoter or enhancer regions of BMP target genes in Lgr5^{+} stem cells.

(**a**) Heatmap of Smad4 bound regions relative to transcription start sites. (**b**) GO analysis of BMP-upregulated genes or -downregulated genes that were bound by Smad4. (**c**) Visualization of the Smad4 binding site and the TCF4 binding site in the first intron of *Lgr5*. The TCF4 binding site was identified based on TCF4 ChIP-seq in intestinal crypts (GEO: GSE65322). (**d**) β-catenin ChIP assays in Lgr5^{+} stem cells with or without 4 hour BMP4 treatment. Data represent mean ± s.e.m of three independent experiments. (**e**) Smad4 ChIP signals at the promoter or enhancer regions of representative cell cycle arrest genes.

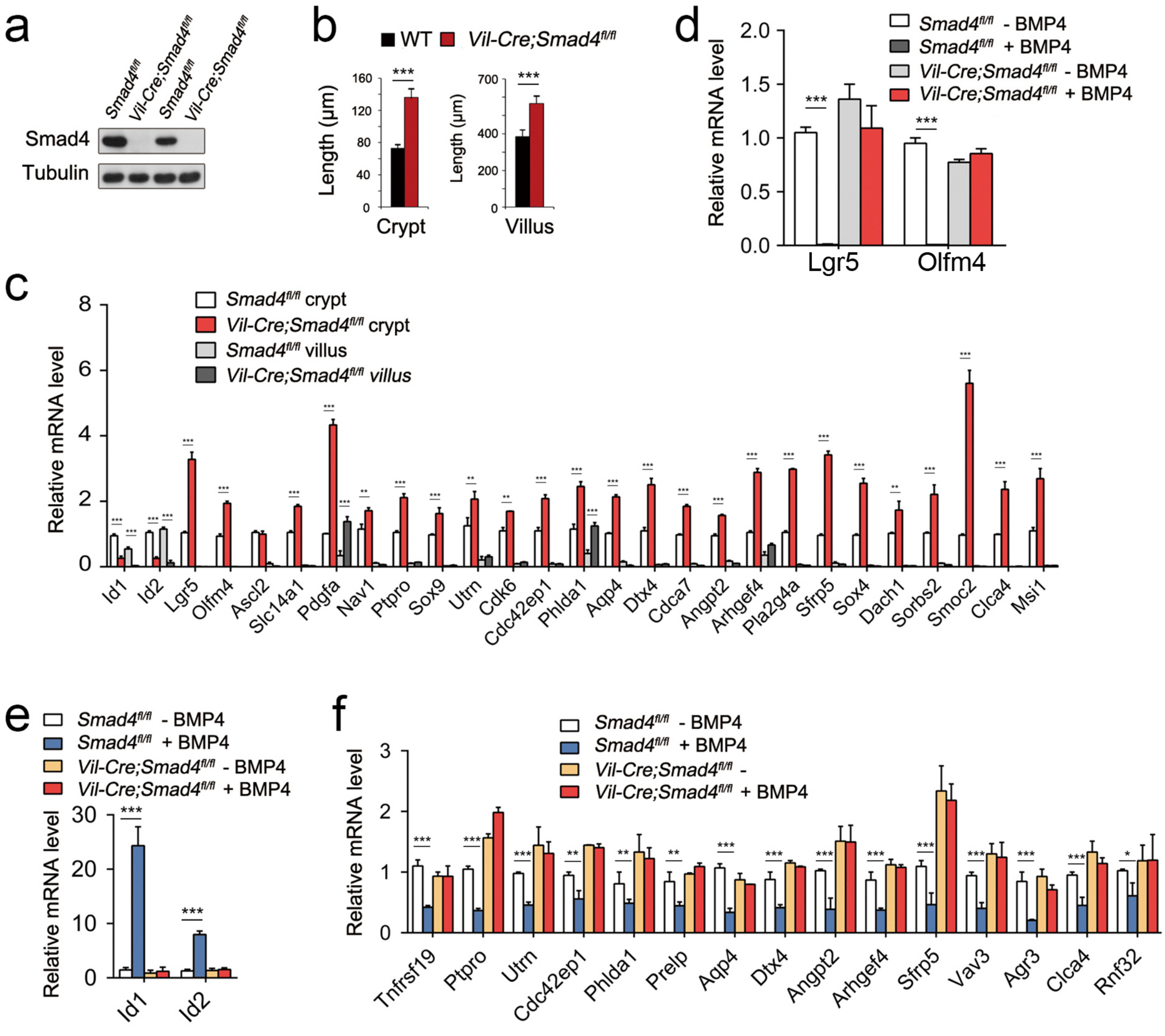

Supplementary Figure 7 Deletion of Smad4 abolishes BMP response in organoids. (**a**) Immunoblots of small intestine epithelium from 1-month old *Smad4*$^{fl/fl}$ and *Vil-cre;Smad4*$^{fl/fl}$ mice. Images are representative of n=3 mice per genotype. (**b**) Quantification of crypt length and villus length in 1-month old *Smad4*$^{fl/fl}$ and *Vil-cre;Smad4*$^{fl/fl}$ mice. Data represent mean ± s.e.m of n=5 mice per genotype. ***P < 0.001 by Student's *t*-tests. (**c**) Quantitative RT-PCR analysis of *Id1*, *Id2* and BMP-regulated signature gene expression in crypts or villus from the mice with indicated genotypes. Data represent mean ± s.e.m of n=5 mice per genotype. **P < 0.01, ***P < 0.001 by Student's *t*-tests. (**d**) Quantitative RT-PCR analysis of *Lgr5*, *Olfm4* and *Ascl2* expression in the organoids treated with or without BMP4 for 36 hours. Data represent mean ± s.e.m of three independent experiments. ***P < 0.001 by Student's *t*-tests. (**e,f**) Quantitative RT-PCR analysis of gene expression in the organoids derived from the mice with indicated genotypes after treated with BMP4 for 4 hours. Data represent mean ± s.e.m of three independent experiments. *P < 0.05, **P < 0.01, ***P < 0.001 by Student's *t*-tests. Scale bars, 50 μm.

Fig. 4c

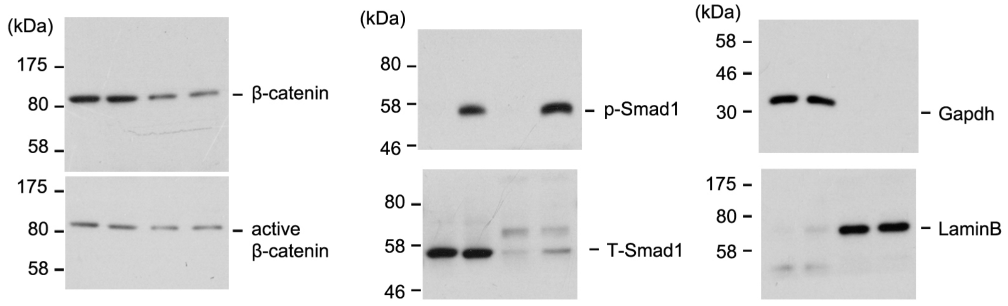

Fig. 5f

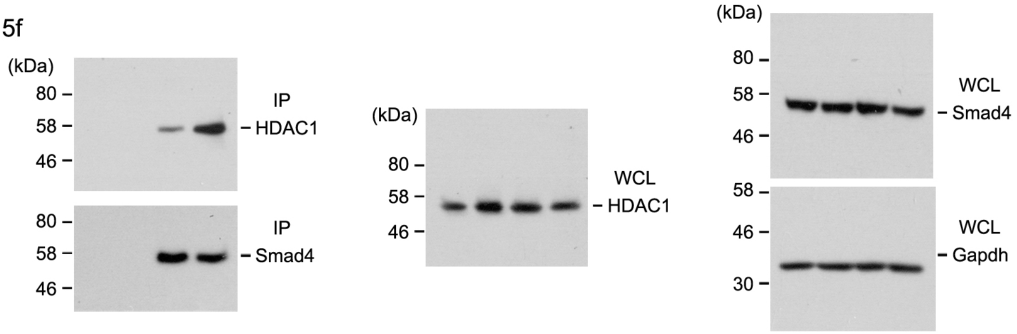

Supplementary Fig. 1c

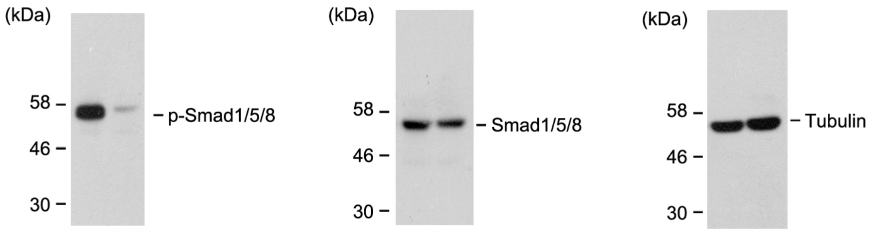

Supplementary Fig. 7a

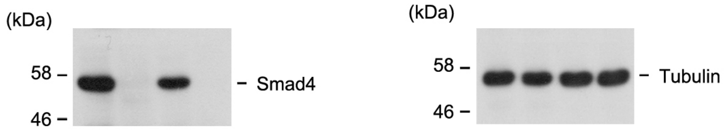

Supplementary Figure 8 Uncropped scans of western blots included in main figures.
